# GhTOPP4aD and GhRAF36 inversely regulate cotton (*Gossypium hirsutum*) response to ABA and salt stress through reversible phosphorylation of GhABI1

**DOI:** 10.1111/pbi.70166

**Published:** 2025-06-08

**Authors:** Pengfei Cao, Lin Zhou, Mingwei Du, Xiaoli Tian, Fangjun Li, Zhaohu Li

**Affiliations:** ^1^ State Key Laboratory of Plant Environmental Resilience, Engineering Research Center of Plant Growth Regulator, Ministry of Education & College of Agronomy and Biotechnology China Agricultural University Beijing China

**Keywords:** salt stress, Type One Protein Phosphatase 4aD (TOPP4aD), ABA insensitive 1 (ABI1), GhRAF36, abscisic acid (ABA)

## Abstract

The post‐translational phosphorylation modification of stress‐related proteins regulated by kinases and phosphatases is one of the crucial regulatory mechanisms for plants in response to salt stress. However, the paired kinases and phosphatases of the same substrate that participate in response to salt tolerance in crops, especially in cotton, remain to be elucidated. Here, we identified GhTOPP4aD as a negative regulator of salt‐stress response in cotton. GhTOPP4aD interacted with Raf‐like kinase 36 (GhRAF36) and ABA Insensitive 1 (GhABI1) respectively, thereby inhibiting the phosphorylation activity of GhRAF36 and directly dephosphorylating GhABI1 to counteract GhRAF36 regulation. The phosphatase activity of GhABI1 was inhibited by GhRAF36‐mediated phosphorylation at two unique residues Thr124 and Ser357 in cotton, whereas it was compromised by GhTOPP4aD. GhTOPP4aD thereby limited ABA signal transduction and orchestrated ABA‐responsive gene expression. Together, modulation of the phosphorylation dynamics of GhABI1 by GhRAF36 kinase and GhTOPP4aD phosphatase constitutes an essential mechanism for ABA response and salt tolerance in cotton.

## Introduction

Cotton is one of the world's leading fibre crops and serves as a pioneer crop in arid and saline‐alkali soils (Flowers *et al*., 2010). Although classified as a salt‐tolerant crop, it is sensitive to salt stress during the germination and seedling stages (Khan *et al*., 2018; Rodriguez‐Uribe *et al*., 2011). Salt stress can cause dehydration and wilting of plants, restrict cell growth and division and disrupt the various biochemical and metabolic processes, resulting in reduced crop quality and yield (Sharif *et al*., 2019; Zhang *et al*., 2014). Therefore, understanding the molecular mechanisms of salt‐stress responses is crucial for cultivating crops that resist salt stress.

High salinity causes osmotic stress and Na^+^ ion toxicity in plants (Hasegawa *et al*., 2000), which triggers an increase in the plant hormone Abscisic Acid (ABA) (Bartels and Sunkar, 2005; Jia *et al*., 2002). ABA can rapidly induce the expression of stress‐responsive genes and trigger multiple physiological responses, such as stomatal closure to reduce water loss in plants (Geiger *et al*., 2009; Johnson *et al*., 2002; Lee *et al*., 2009). As the negative regulators, Protein Phosphatase 2Cs (PP2Cs) are key components of the ABA signalling pathway. The phosphatase activity of PP2Cs is inhibited by ABA‐bound PYR1/PYLs/RCAR receptors, promoting the downstream transduction of ABA signals (Ma *et al*., 2009; Park *et al*., 2009). Arabidopsis Enhancer of ABA Co‐Receptor1 (EAR1) or Rho‐like small GTPase ROP11/10 binds to members of the PP2Cs family, such as ABA Insensitive 1/2 (ABI1/2), thereby enhancing the phosphatase activity of PP2Cs and negatively regulating the ABA signalling pathway (Wang *et al*., 2018; Yu *et al*., 2012). Additionally, Transmembrane Kinase Protein 4 (TMK4) phosphorylates ABI2 and enhances its phosphatase activity in Arabidopsis (Li *et al*., 2021a).

Raf‐like protein kinases (RAFs) are classified into four B and seven C subgroups based on sequence similarity (Ichimura *et al*., 2002). The B2/B3 subgroup RAF kinases are essential for the activation of SnRK2 upon ABA or osmotic stress (Lin *et al*., 2020; Soma *et al*., 2023). High‐order mutants such as OK^130^‐null and OK‐quindec reveal the critical roles of RAFs in osmotic stress tolerance, ABA responses, as well as growth and development in Arabidopsis (Lin *et al*., 2020). The two members of the C subgroup, RAF22 and RAF36, are direct substrates of SnRK2s and negatively regulate ABA signalling in Arabidopsis (Kamiyama *et al*., 2021; Sun *et al*., 2022). Moreover, RAF22, similar to TMK4 kinase, can directly phosphorylate ABI1 and enhance the phosphatase activity of ABI1, thereby mediating the dynamic balance between plant growth and drought stress responses (Sun *et al*., 2022). However, the impact of phosphatases related to ABI on its phosphatase activity remains a mystery.

Eukaryotic protein phosphatases are classified into several types based on substrate specificity, catalytic mechanism and sensitivity to inhibitors. These include serine/threonine‐specific Phosphoprotein Phosphatase (PPP), Metal ion‐dependent Protein Phosphatase (PPM), Dual Specificity Phosphatase (DSP) and Phosphotyrosine Phosphatase (PTP) (Farkas *et al*., 2007). Protein Phosphatase 1 (PP1) belongs to the PPP family and is broadly involved in various plant processes such as light signalling, growth and development and hormone regulation (Ceulemans and Bollen, 2004; Farkas *et al*., 2007). Among its members, TOPP4 dephosphorylates the DELLA protein in the gibberellin (GA) signalling pathway, promoting the downstream transduction of GA signals and regulating plant growth and development in Arabidopsis (Qin *et al*., 2014). Phytochrome B induces the phosphorylation of Phytochrome‐Interacting Factors 5 (PIF5), which is dephosphorylated by TOPP4, allowing it to accumulate and regulate photomorphogenesis (Yue *et al*., 2016). TOPP4 also participates in the development of leaf epidermal cells by regulating the phosphorylation state of Pin‐Formed Protein 1 (PIN1) in Arabidopsis (Guo *et al*., 2015). Although Arabidopsis Type One Protein Phosphatase 1 (TOPP1) interacts with At Inhibitor‐2 (AtI‐2) to negatively regulate the ABA signalling pathway, and *Attopp1* mutants are sensitive to both ABA and salt stress (Hou *et al*., 2016). The roles of TOPPs in cotton salt‐stress response remain largely unknown. Here, we report that salt stress relieves the inhibition of GhTOPP4aD on GhRAF36 kinase activity towards GhABI1 to inhibit GhABI1 phosphatase activity, thereby promoting the downstream transduction of ABA signals. Thus, our study identified GhRAF36 and GhTOPP4aD as the paired kinase and phosphate of GhABI1 to regulate ABA and salt‐stress responses in cotton.

## Results

### GhTOPP4aD of the PP1 family is identified as a negative regulator of cotton salt tolerance

**Figure 1:**
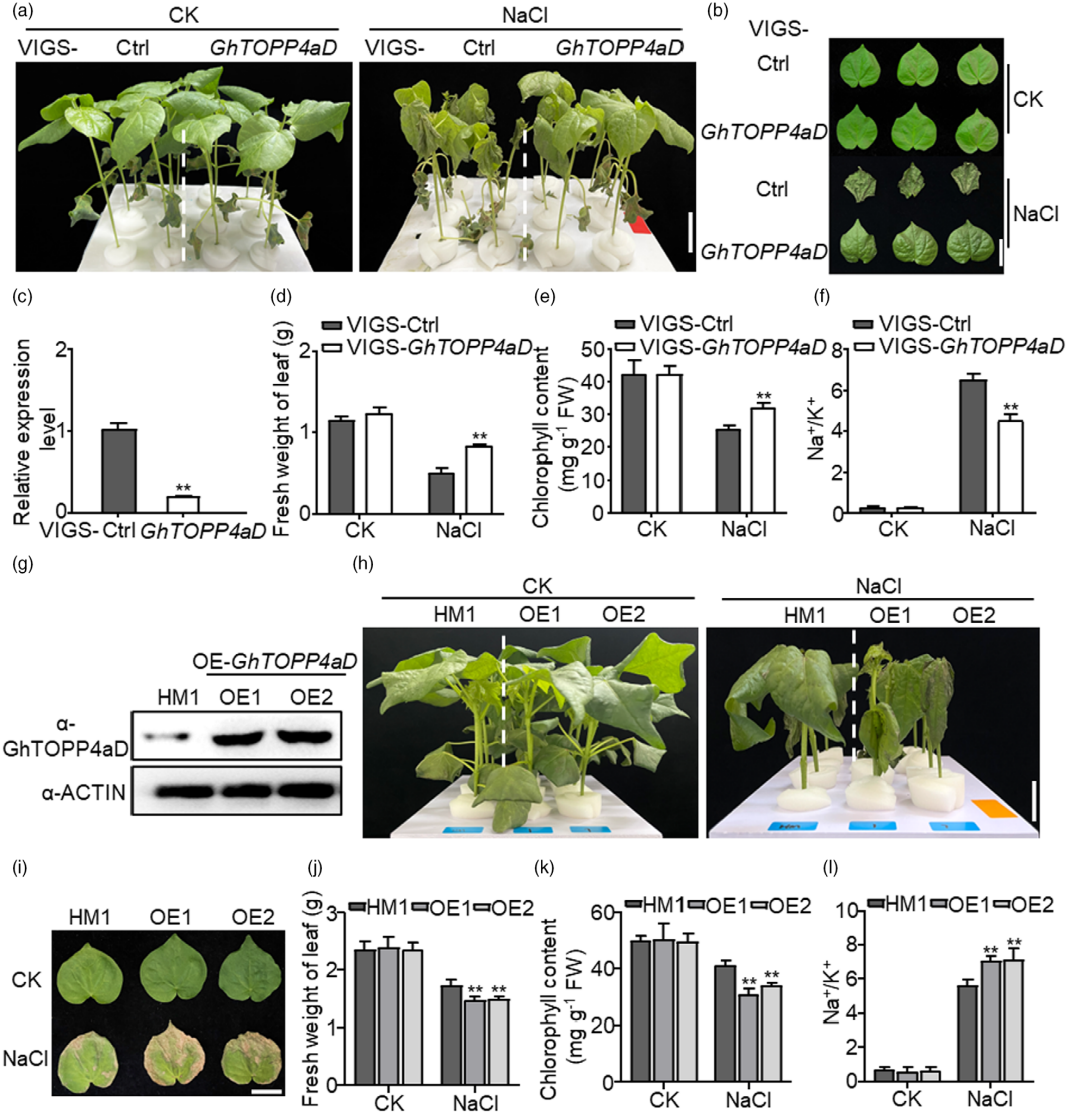
*GhTOPP4aD* is a negative regulator in cotton salt response. (a)Silencing *GhTOPP4aD* enhances cotton salt tolerance. Fourteen‐day‐old silenced plants were subjected to 300 mM NaCl for 3 d. The dashed lines separate different VIGS groups. Bar = 3 cm. (b) Phenotype of the leaves of VIGS plants after 3 days of NaCl treatment. Bar = 3 cm. (c) Silencing efficiency of *GhTOPP4aD* in VIGS‐*GhTOPP4aD* plants. The leaf samples from VIGS‐Ctrl and VIGS‐*GhTOPP4aD* cotton seedlings were collected to detect the expression of *GhTOPP4aD* without NaCl treatment by real‐time quantitative PCR (RT‐qPCR). GhActin9 was used as the internal control. The data are shown as means ± SD from three independent repeats (*n* = 3; **P < 0.01, Student's *t*‐test). (d) The fresh weight of leaves in VIGS‐Ctrl and VIGS‐*GhTOPP4aD* plants without or with NaCl treatment. The data are shown as means ±SD from three independent repeats (*n* = 3; **P < 0.01, Student's *t*‐test). (e) The chlorophyll content in VIGS‐Ctrl and VIGS‐*GhTOPP4aD* plants without or with NaCl treatment. The data are shown as means ± SD from three independent repeats (*n* = 3; **P < 0.01, Student's *t*‐test). (f) Na^+^/K^+^ of VIGS‐Ctrl and VIGS‐*GhTOPP4aD* under CK and NaCl treatment. The data are shown as means ± SD from three independent repeats (*n* = 3; **P < 0.01, Student's *t*‐test). (g) Determination of TOPP4aD protein levels in HM1 and OE‐*GhTOPP4aD* plants with NaCl treatment at indicated time points using Anti‐GhTOPP4aD antibodies. GhACTIN was used as a loading control. (h) OE‐*GhTOPP4aD* plants are more salt stress sensitive than the recipient plants (HM1). Fourteen‐day‐old transgenic plants were subjected to 300 mM NaCl for 3 d. Bar = 3 cm. (i) Phenotypes of the leaves in OE‐*GhTOPP4aD* plants after 3 days of salt‐stress treatment. Bar = 3 cm. (j) The fresh weight of leaves in HM1 and OE‐*GhTOPP4aD* without or with NaCl treatment. The data are shown as means ± SD from three independent repeats (*n* = 3; **P < 0.01, Student's *t*‐test). (k) The chlorophyll content in HM1 and OE‐*GhTOPP4aD* without or with NaCl treatment. The data are shown as means ± SD from three independent repeats (*n* = 3; **P < 0.01, Student's *t*‐test). (l) Na^+^/K^+^ in HM1 and OE‐*GhTOPP4aD* without or with NaCl treatment. The data are shown as means ± SD from three independent repeats (*n* = 3; **P < 0.01, Student's *t*‐test).

**Figure 2:**
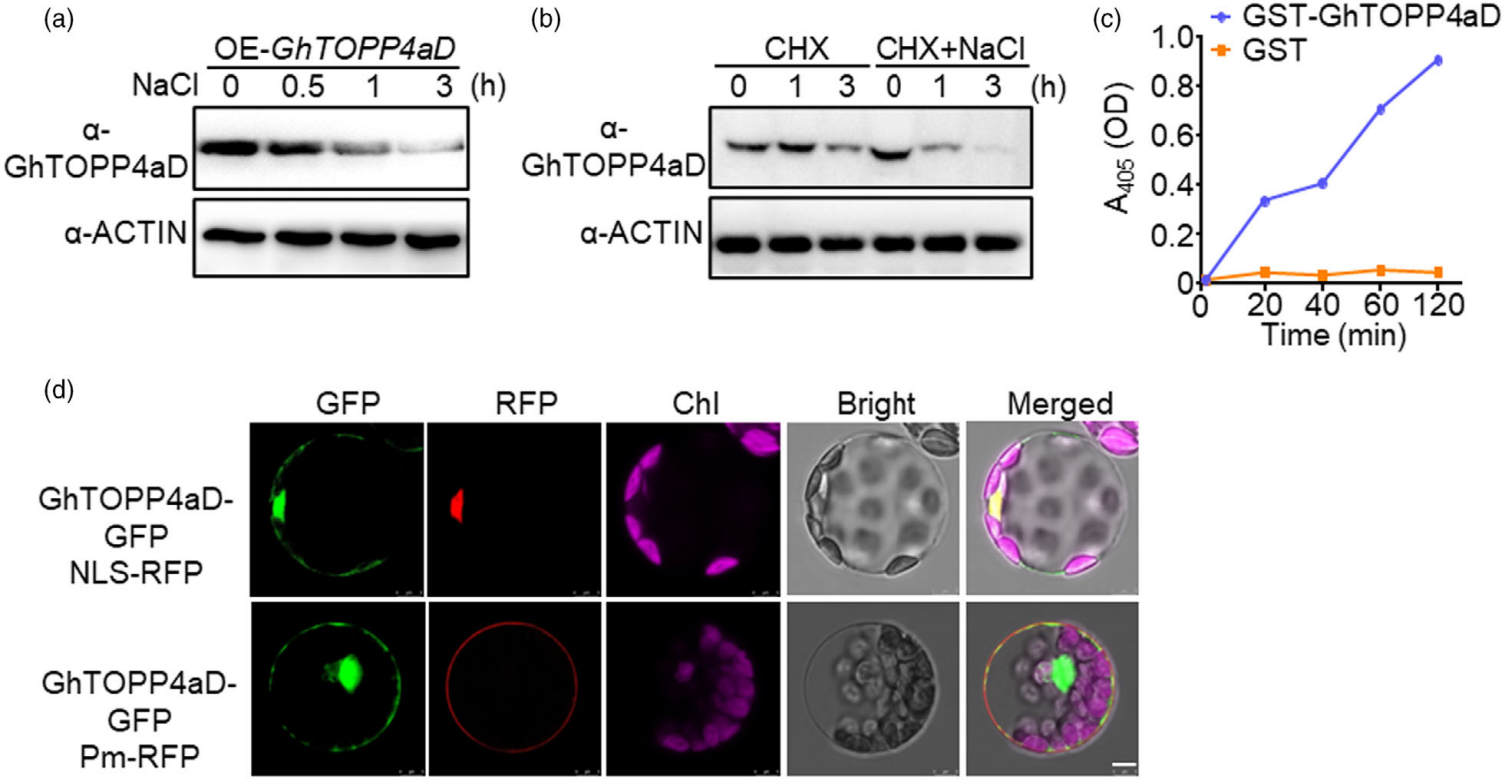
Salt stress promotes the degradation of GhTOPP4aD phosphatase. (a) The protein abundance of GhTOPP4aD was determined by immunoblotting using anti‐GhTOPP4aD antibodies. Fourteen‐day‐old OE‐*GhTOPP4aD* seedlings were treated with 300 mM NaCl for the indicated time. GhACTIN was used as a loading control. (b) Salt stress promotes GhTOPP4aD degradation. Fourteen‐day‐old OE‐*GhTOPP4aD* seedlings were treated with 150 μM CHX or 150 μM CHX plus 300 mM NaCl for the indicated time. Proteins were extracted and used for immunoblotting analysis with anti‐GhTOPP4aD antibodies. GhACTIN was used as a loading control. (c) Phosphatase activity of GhTOPP4aD is enhanced with increasing time points. About 2 μg GhTOPP4aD or GST protein was used as phosphatase. The phosphatase activity of GhTOPP4aD was measured as absorbance at 405 nm (A_405_). (d) GhTOPP4aD localizes in both cytoplasm and nucleus. NLS‐RFP and GhTOPP4aD‐GFP were co‐expressed in cotton protoplasts for 12 h; GFP signal was examined using a confocal microscope. Bright field and green fluorescence images were merged overlay. Scale bars = 5 μm.

Post‐transcriptional reversible phosphorylation modification regulated by protein kinases and phosphatases is a crucial regulatory mechanism for plants in response to stress signals (Wang *et al*., 2007). PP1 is an important phosphatase regulating stress responses. However, the roles of PP1 in plant response to salt tolerance, especially in cotton, remain unrevealed. In Arabidopsis, there are nine isoforms of PP1, which are named Type One Protein Phosphatases (TOPPs) (Liu *et al*., 2020). In our previous work, we identified the cotton orthologues sharing the closest amino acid sequence similarity with Arabidopsis TOPP1‐9 through blasting in the *G. hirsutum* NBI proteins database (www.cottongen.org/tools/blast) (Figure S1a). Regarding the gene nomenclature, the lowercase letters (a) after the cotton gene are used to distinguish different genes within the same evolutionary branch, while the uppercase letters A and D that appear last indicate different subgenomes. After screening for salt tolerance phenotypes using VIGS to silence *GhTOPPs*, we found that VIGS‐*GhTOPP4aD* plants with gene expression decreased by 81.24% exhibited more flattened and vigorous leaves compared to VIGS‐Ctrl plants (Figure 1a–c). As *GhTOPP4aA* (Gh_A10G2014) and *GhTOPP4aD* (Gh_D10G2504) share 98.74% amino acid sequence similarity and possess conserved VIGS fragments for gene silencing, we speculated that they probably have similar protein function and play a role together in salt tolerance regulation. Consistently, silencing *GhTOPP4aD* increased the fresh weight and chlorophyll content by 68.35% and 36.44%, respectively, in leaves under salt stress compared to those in control plants (Figure 1d,e). Maintaining the balance of sodium‐to‐potassium ratio (Na^+^/K^+^) is an important physiological characteristic of plant salt tolerance (Rubio *et al*., 1995). Next, we detected Na^+^ and K^+^ contents in VIGS‐*GhTOPP4aD* plants and found that silencing *GhTOPP4aD* significantly reduced Na^+^ but increased K^+^ content by 19.29% and 28.64% (Figure S1b,c), thus maintaining significantly lower Na^+^/K^+^ in cotton leaves under salt stress (Figure 1f). To clarify the role of *GhTOPP4aD* in cotton salt tolerance, we overexpressed *GhTOPP4aD* in cotton under the control of the 35S promoter and obtained two independent homozygous lines (1# and 2#) with induced protein expression of GhTOPP4aD by using anti‐GhTOPP4aD antibodies (Figure 1g). Contrary to the salt‐tolerant phenotype of VIGS‐*GhTOPP4aD*, OE‐*GhTOPP4aD* plants showed severe leaf wilting, less fresh weight of leaf and chlorophyll content compared to those in recipient (HM1) under salt stress (Figure 1h–k). Moreover, two overexpression of *GhTOPP4aD* lines showed significantly increased Na^+^ content by 8.56% and 16.67% and Na^+^/K^+^ by 26.07% and 29.74% in leaves, while no significant difference of K^+^ content compared with those in HM1 (Figures 1l and S1d,e). We also noticed that the protein abundance of GhTOPP4aD gradually decreased at indicated time points after NaCl treatment (Figure 2a,b). The results demonstrated that GhTOPP4aD, a protein phosphatase, negatively regulates cotton salt‐stress response.

Meanwhile, we purified GST‐GhTOPP4aD recombinant protein from *E. coli* BL21 strain to detect its phosphatase activity *in vitro* using pyro‐nitrophenyl phosphate (pNPP) as a substrate. The GhTOPP4aD proteins exhibited strong protein phosphatase activity by the hydrolysis of pNPP into p‐nitrophenol, a chromogenic product with absorbance at 405 nm (Figure 2c). To further understand the GhTOPP4aD protein function, we fused it with green fluorescent protein (GFP) to perform the transient expression in cotton protoplasts and found that GhTOPP4aD was mainly localized in the nucleus, with a small amount located in the cytoplasm (Figure 2d).

### GhTOPP4aD interacts with and dephosphorylates GhRAF36 in cotton

**Figure 3:**
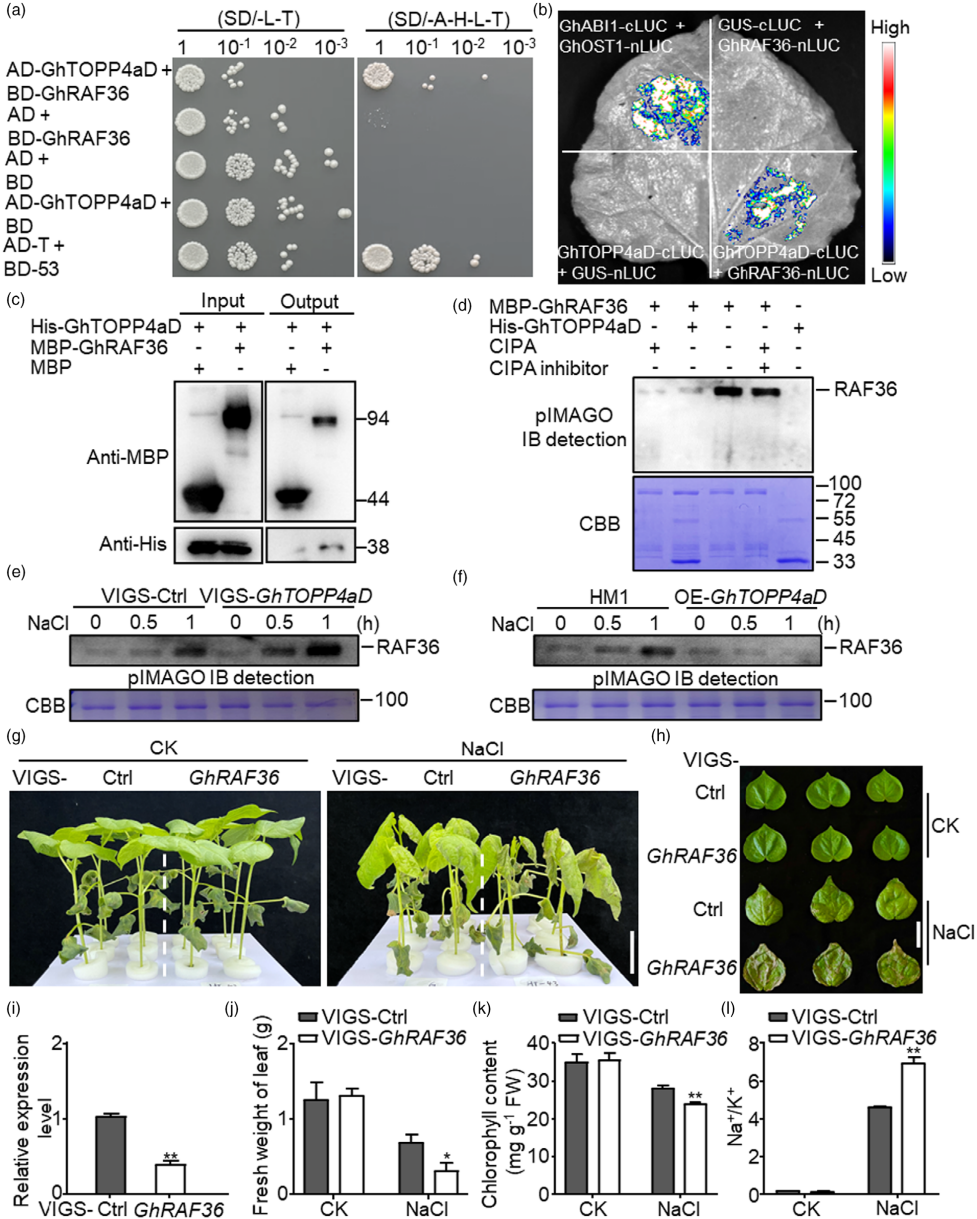
GhTOPP4aD physically interacts with GhRAF36 and inhibits its kinase activity. (a) Yeast two‐hybrid (Y2H) assay showing the interaction between GhTOPP4aD and GhRAF36. SD/‐L‐T, synthetic medium without Trp and Leu; SD/‐A‐H‐L‐T, synthetic medium without Trp, Leu, His and Ade. DNA binding domain (BD) and activation domain (AD) were used as empty controls. (b) LUC complementation imaging (LCI) assay showing the interaction between GhTOPP4aD and GhRAF36. Representative images of *N. benthamiana* leaves 48 h after infiltration are shown. GhOST1‐nLUC and cLUC‐GhABI1 served as positive controls. The bar showing red to blue indicates luciferase signal intensity from high to low. (c) Analysis of the interaction between GhTOPP4aD and GhRAF36 *in vitro* by pull‐down assay. Recombinant MBP‐GhRAF36 or His‐GhTOPP4aD protein was purified from *E. coli*. Protein was detected with anti‐His and anti‐MBP antibodies. (d) GhTOPP4aD inhibits GhRAF36 kinase activity *in vitro*. About 2 μg recombinant purified MBP‐GhRAF36 was incubated with 4 μg His‐GhTOPP4aD or CIPA to perform *in vitro* kinase assay. The proteins were separated by 12.5% SDS‐PAGE. (e, f) GhRAF36 kinase activity is enhanced in VIGS‐*GhTOPP4aD* and reduced in OE‐*GhTOPP4aD* plants. The total proteins were extracted as kinases from leaves of 14‐day‐old VIGS plants or overexpression line leaves with or without 300 mM NaCl treatment. Pre‐dephosphorylated GhRAF36 as a substrate was incubated with total proteins in kinase reaction buffer for 30 min at 30 °C and then separated by SDS‐PAGE. (g, h) Silencing *GhRAF36* compromises salt tolerance in cotton. Fourteen‐day‐old silenced plants were subjected to 250 mM NaCl for 3 d. The dashed lines separate different VIGS groups. Bar = 3 cm. (i) Silencing efficiency of *GhRAF36* in VIGS‐*GhRAF36* plants. The leaf samples from VIGS‐Ctrl and VIGS‐*GhRAF36* cotton seedlings were collected to detect the expression level of *GhRAF36* without NaCl treatment by RT‐qPCR. GhActin9 was used as the internal control. The data are shown as means ± SD from three independent repeats (*n* = 3; **P < 0.01, Student's *t*‐test). (j) The fresh weight of leaves in VIGS‐Ctrl and VIGS‐*GhRAF36* without or with NaCl treatment. The data are shown as means ± SD from three independent repeats (*n* = 3; *P < 0.05, **P < 0.01, Student's *t*‐test). (k) The chlorophyll content in VIGS‐Ctrl and VIGS‐*GhRAF36* without or with NaCl treatment. The data are shown as means ± SD from three independent repeats (*n* = 3; **P < 0.01, Student's *t*‐test). (l) Na^+^/K^+^ in VIGS‐Ctrl and VIGS‐*GhRAF36* without or with NaCl treatment. The data are shown as means ±SD from three independent repeats (*n* = 3; **P < 0.01, Student's *t*‐test). The experiments were performed three times (a–l) with similar results.

To elucidate the underlying molecular mechanism of GhTOPP4aD regulating cotton salt‐stress response as a protein phosphatase, we screened a cotton yeast‐two‐hybrid (Y2H) cDNA library, using GhTOPP4aD as a ‘bait’. Among the candidate proteins that interact with GhTOPP4aD (Table S1), we noticed a group C RAF interacted strongly with GhTOPP4aD in yeast (Figure 3a), which shared 59.1% amino acid sequence similarity with AtRAF36 (AT5G58950). To further confirm the interaction between GhTOPP4aD and GhRAF36 (Gh_D05G1535), we performed LUC complementation imaging (LCI) and bimolecular fluorescence complementation (BiFC) assays and found that GhRAF36 and GhTOPP4aD also interacted in *N. benthamiana* (Figures 3b and S2a). Moreover, the recombinant proteins of GhRAF36 and GhTOPP4aD directly interacted *in vitro* (Figure 3c).

In Arabidopsis, ABA‐activated SnRK2s phosphorylate RAF36 and promote RAF36 degradation, leading to stronger ABA responses under abiotic stress (Kamiyama *et al*., 2021). Here, we found that GhRAF36 displayed strong autophosphorylation activity *in vitro*, which was significantly inhibited when incubated with Calf Intestine Phosphatase Alkaline (CIPA) (Figure 3d). Moreover, the autophosphorylation of RAF inhibited by CIPA was significantly restored by a CIPA inhibitor (Figure 3d). To detect whether GhTOPP4aD could affect the autophosphorylation of GhRAF36, we performed a dephosphorylation assay with His‐GhTOPP4aD as a phosphatase and MBP‐GhRAF36 as substrates and found that similar to CIPA treatment, GhTOPP4aD could also strongly abolish the autophosphorylation of GhRAF36 (Figure 3d). In addition, we incubated MBP‐GhRAF36 protein with the total proteins extracted from HM1 and OE‐*GhTOPP4aD* plants without salt treatment and found that GhTOPP4aD could significantly inhibit the kinase activity of GhRAF36 under normal conditions (Figure S2b), indicating that GhRAF36 is probably a dephosphorylation target of GhTOPP4aD.

Next, we performed an *in vitro* phosphorylation assay using the extracted total proteins from VIGS‐Ctrl, VIGS‐*GhTOPP4aD*, HM1 and OE‐*GhTOPP4aD* with or without NaCl treatment as the kinase and MBP‐GhRAF36 with CIPA pre‐treatment as the substrate. The data showed that salt could induce GhRAF36 phosphorylation, and silencing *GhTOPP4aD* further enhanced salt‐induced GhRAF36 phosphorylation (Figure 3e), while overexpression of *GhTOPP4aD* exhibited the opposite trends (Figure 3f). Together, the results suggest that GhTOPP4aD interacts with and inhibits the autophosphorylation of GhRAF36 by directly dephosphorylating it.

To explore the physiological functions of *GhRAF36* under salt stress, we generated VIGS‐*GhRAF36* plants to detect its response to salt stress. Compared with the control plants, VIGS‐*GhRAF36* plants with gene expression decreased by 64.65% exhibited more severe leaf wilting and enhanced salt sensitivity in cotton (Figure 3g–i), with less fresh weight of leaf and chlorophyll content (Figure 3j,k). Furthermore, silencing *GhRAF36* significantly increased Na^+^ but not K^+^ content in cotton leaves upon salt treatment (Figure S2c,d), leading to a higher Na^+^/K^+^ ratio compared to that in VIGS‐Ctrl plants (Figure 3l). The results indicate that GhRAF36 is a positive regulator in cotton salt‐stress response.

### GhRAF36 interacts with and phosphorylates GhABI1

**Figure 4:**
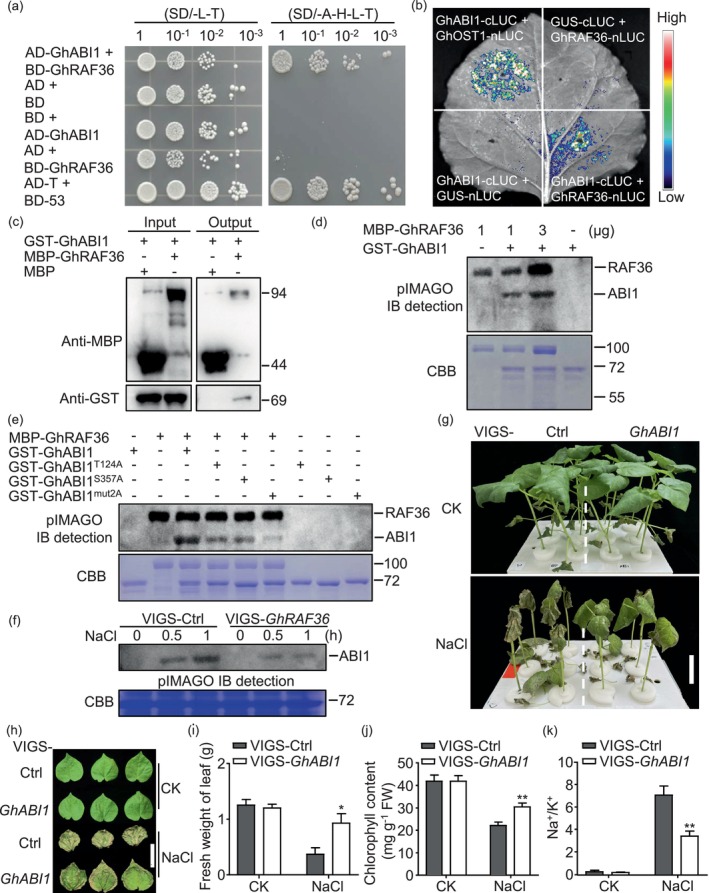
GhRAF36 physically interacts with GhABI1 and phosphorylates GhABI1. (a) Yeast two‐hybrid (Y2H) assay showing the interaction between GhRAF36 and GhABI1. SD/‐L‐T, synthetic medium without Trp and Leu; SD/‐A‐H‐L‐T, synthetic medium without Trp, Leu, His, and Ade. BD and AD were used as empty controls. (b) GhRAF36 interacts with GhABI1 by LCI assay. Representative images of *N. benthamiana* leaves 48 h after infiltration are shown. The bar showing red to blue indicates luciferase signal intensity from high to low. (c) GhRAF36 interacts with GhABI1 *in vitro* by pull‐down assay. Recombinant MBP‐GhRAF36 or GST‐GhABI1 protein was purified from *E. coli*. Protein was detected with anti‐GST and anti‐MBP antibodies. (d) GhRAF36 phosphorylates GhABI1 *in vitro*. Purified recombinant GST‐GhABI1 (2 μg) was incubated with MBP‐GhRAF36 (2, 6 μg) to perform an *in vitro* kinase assay. The proteins were separated by 10% SDS‐PAGE. (e) Phosphorylation of GhABI1 and its mutated variants by GhRAF36 *in vitro*. Purified recombinant GST‐GhABI1, GST‐GhABI1T124A and GST‐GhABI1S357A were incubated with MBP‐GhRAF36 to perform an *in vitro* kinase assay. The proteins were separated by 10% SDS‐PAGE. (f) *In vitro* phosphorylation assay indicated the phosphorylation of GhABI1 without or with salt‐stress treatment in VIGS plants. The total proteins were extracted as kinases from 14‐day‐old VIGS‐Ctrl and VIGS‐*GhRAF36* cotton leaves with or without 300 mM NaCl treatment. GhABI1 as a substrate was incubated with total proteins in kinase reaction buffer for 30 min at 30 °C and then separated by SDS‐PAGE. (g, h) The phenotypes of VIGS‐Ctrl and VIGS‐*GhABI1* under salt stress. Fourteen‐day‐old silenced plants were subjected to 300 mM NaCl for 3 d. The dashed lines separate different VIGS groups. Bar = 3 cm. (i–k) The fresh weight of leaves in VIGS‐Ctrl and VIGS‐*GhABI1* without or with NaCl treatment (i). The chlorophyll content in VIGS‐Ctrl and VIGS‐*GhABI1* without or with NaCl treatment (j). Na^+^/K^+^ in VIGS‐Ctrl and VIGS‐*GhABI1* without or with NaCl treatment (k). The data are shown as means ± SD from three independent repeats (*n* = 3; **P < 0.01, Student's *t*‐test). The experiments were performed three times (a–k) with similar results.

To further identify the downstream phosphorylation targets of GhRAF36, we screened a cotton Y2H cDNA library using GhRAF36 as a ‘bait’. It is noteworthy that cotton ABA insensitive 1 (GhABI1, Gh_D13G2089) sharing 42.7% amino acid sequence identity with AtABI1, a key phosphatase of ABA signalling in Arabidopsis, was identified among the candidate proteins interacting with GhRAF36 (Table S2). Next, we verified that GhABI1 interacted with GhRAF36 in yeast and *N. benthamiana* leaves by LCI and BiFC assay (Figures 4a,b and S3a). Moreover, the recombinant proteins of MBP‐GhRAF36 and GST‐GhABI1 could directly interact *in vitro* (Figure 4c).

To detect whether GhRAF36 can phosphorylate GhABI1, we performed a phosphorylation assay with MBP‐GhRAF36 as a kinase and GST‐GhABI1 as a substrate and found that GhRAF36 directly phosphorylated GhABI1 in a dosage‐dependent manner (Figure 4d). Next, we conducted liquid chromatography–tandem mass spectrometry (LC–MS/MS) analysis and identified two putative phosphorylation sites, which are threonine at position 124 (T124) and serine at position 357 (S357), respectively, in the phosphatase catalytic domain of GhABI1 (Figure S3b,c). Mutation of T124 or S357 to Ala (A) significantly reduced phosphorylation of GhABI1 by GhRAF36, which was further inhibited by double mutation of S124 and S357 (Figure 4e). However, we found that these two phosphorylation sites on GhABI1 were not conserved in cotton and its orthologues from other species (Figure S3d), suggesting that the phosphorylation of T124 and S357 sites on GhABI1 by GhRAF36 could be specific to cotton. We also examined the phosphorylation of GhABI1 in VIGS‐Ctrl and VIGS‐*GhRAF36* plants and found that salt could induce GhABI1 phosphorylation, which was significantly inhibited by silencing *GhRAF36* (Figure 4f). These results indicate that GhRAF36 might regulate GhABI1 through direct phosphorylation.

AtABI1 is a negative modulator in salt‐stress response (Krzywinska *et al*., 2016; Ohta *et al*., 2003). Here, we utilized VIGS to silence *GhABI1* in cotton and found that VIGS‐*GhABI1* plants exhibited significantly stronger salt‐stress tolerance compared to VIGS‐Ctrl plants (Figures 4g,h, and S3e). Moreover, the fresh weight of leaf, chlorophyll content and K^+^ content of VIGS‐*GhABI1* were significantly higher than those of VIGS‐Ctrl (Figures 4i,j and S3f), while the Na^+^ content and Na^+^/K^+^ were lower (Figures 4k and S3g). The results suggest that ABI1 is a negative player in salt‐stress response, which is conserved in cotton and Arabidopsis.

### GhRAF36 inhibits the phosphatase activity of GhABI1 through phosphorylation

**Figure 5:**
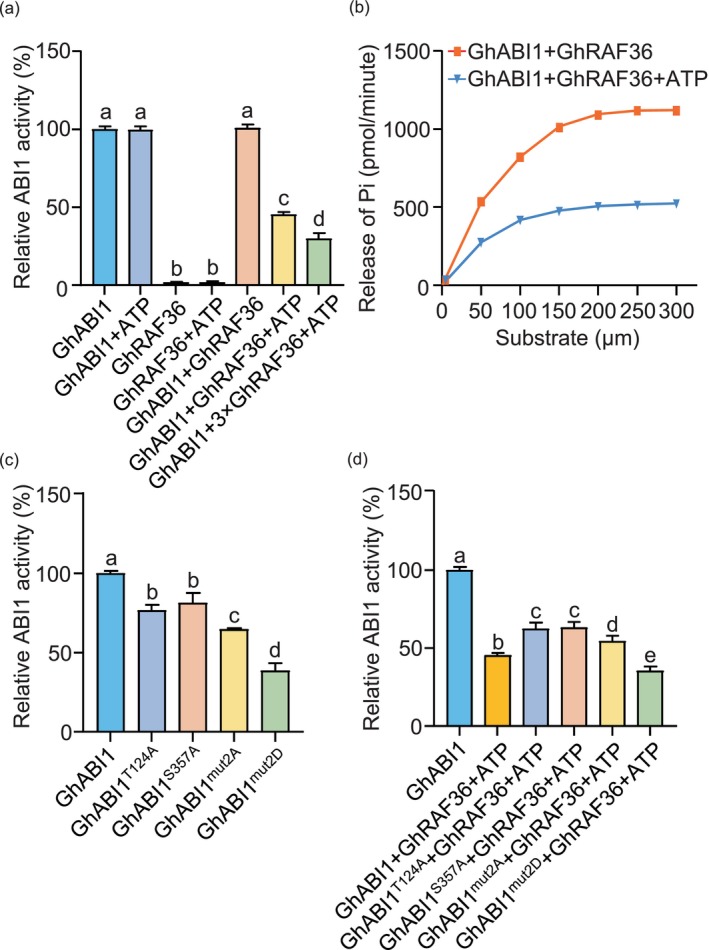
GhRAF36 inhibits GhABI1 phosphatase activity via phosphorylation. (a) GhRAF36 phosphorylates GhABI1 and inhibits its phosphatase activity. Recombinant MBP‐GhRAF36 or GST‐GhABI1 was used for the phosphatase activity assay. The data are shown as means ± SD from three independent repeats (*n* = 3; **P < 0.01, Student's *t*‐test). (b) Kinetic‐dependent curve of GhABI1 phosphatase activity with or without ATP. The substrate concentrations were 50, 100, 150, 200, 250 and 300 μmol/L. GhABI1 phosphatase activity of indicated concentrations was measured. The data are shown as means ± SD from three independent repeats (*n* = 3; **P < 0.01, Student's *t*‐test). (c, d) Phosphatase activity of GhABI1 and its mutant proteins (GhABI1^T124A^, GhABI1^S357A^, GhABI1^mut2A^ and GhABI1^mut2D^) was determined. The data are shown as means ± SD from three independent repeats (*n* = 3; **P < 0.01, Student's *t*‐test).

The phosphorylation modification of protein phosphatase by serine/threonine protein kinase can alter its phosphatase activity (Dohadwala *et al*., 1994). This alteration in activity depends on the phosphorylation of different sites on the phosphatase. Such as AtTMK1 inhibits AtABI2 phosphatase activity by phosphorylating T321 (Yang *et al*., 2021), while AtTMK4 enhances the phosphatase activity of AtABI2 by phosphorylating Ser139, Ser140 and Ser266 (Li *et al*., 2021a). Here, we hypothesized that GhRAF36‐mediated phosphorylation might regulate the phosphatase activity of GhABI1. Thus, we incubated GhABI1 in the presence or absence of ATP with different dosages of GhRAF36 to measure the phosphatase activity of GhABI1. The phosphatase activity of GhABI1 in the absence of the GhRAF36 was set to 100%. GhRAF36 without ATP did not alter the phosphatase activity of GhABI1, but in the presence of both, the phosphatase activity of GhABI1 was significantly reduced (Figure 5a). We also performed kinetic analysis and found that the GhABI1 phosphatase activity was gradually increased in a dosage‐dependent manner and GhRAF36 reduced this kinetic trend (Figure 5b). Meanwhile, we mutated the two phosphorylation sites of GhABI1 to A or Asp (D) to mimic inactive or phosphorylated GhABI1, respectively, and found the phosphatase activity of GhABI1^T124A^ and GhABI1^S357A^ was reduced. Simultaneous mutation of both sites to A or D can further decrease the phosphatase activity of GhABI1, with the reduction being more significant when both sites were mutated to D (Figure 5c). Next, we examined the effect of GhRAF36 on the phosphatase activity of GhABI1 mutant proteins and found that GhABI1^T124A^ and GhABI1^S357A^ significantly compromised the inhibitory effect of GhRAF36 on the phosphatase activity of GhABI1. Consistently, double mutant GhABI1^T124AS357A^ with lower phosphatase activity than GhABI1^T124A^ and GhABI1^S357A^ also significantly suppressed the inhibitory effect of GhRAF36 on its phosphatase activity (Figure 5c,d), indicating that there probably exist other potential phosphorylation sites regulated by GhRAF36 within the catalytic active centre of GhABI1 phosphatase. Intriguingly, the phosphorylated mimic double mutant GhABI1^T124DS357D^ could further enhance the inhibitory effect of GhRAF36 on GhABI1 phosphatase activity (Figure 5d). Together, the results suggest that GhRAF36 inhibits the phosphatase activity of GhABI1 through the phosphorylation at T124 and S357 residues.

**Figure 6:**
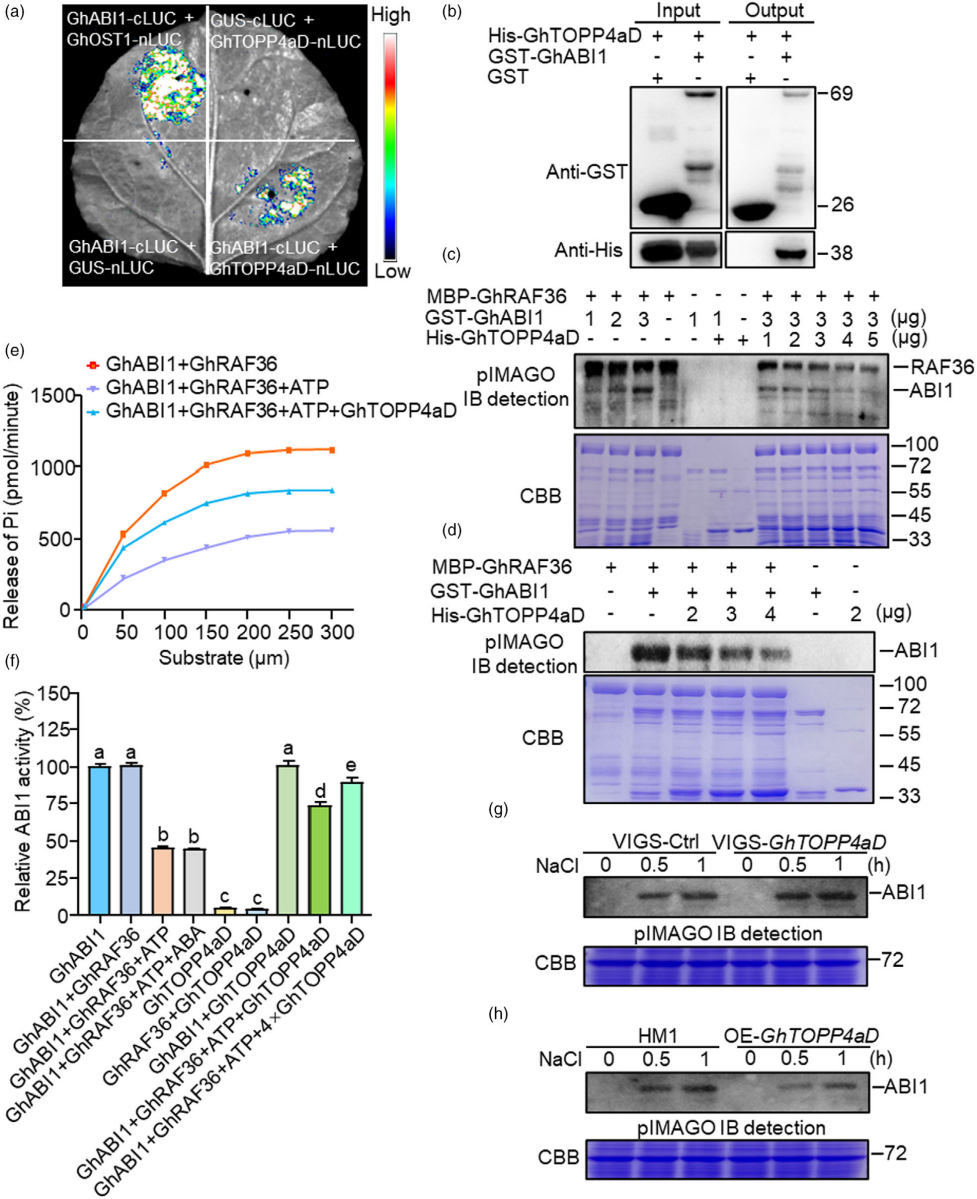
GhRAF36 and GhTOPP4aD inversely regulate the phosphatase activity of GhABI1 by reverse phosphorylation regulation. (a) GhTOPP4aD interacts with GhABI1 by LCI assays. Representative images of *N. benthamiana* leaves after infiltration for 48 h were shown. The bar showing red to blue indicates luciferase signal intensity from high to low. (b) GhTOPP4aD interacts with GhABI1 by pull‐down assay. Recombinant GST‐GhABI1 or His‐GhTOPP4aD proteins were purified from *E. coli*. Protein was detected with anti‐His and anti‐GST antibodies. (c) GhTOPP4aD compromises the phosphorylation of GhABI1 by GhRAF36. Recombinant proteins of MBP‐GhRAF36 (3 μg) and a gradient concentration of His‐GhTOPP4aD (1–5 μg) or GST‐GhABI1 were incubated for the kinase assay at 30 °C for 30 min and separated by 12.5% SDS‐PAGE, respectively. (d) GhTOPP4aD directly dephosphorylates GhABI1 regulated by GhRAF36. Recombinant proteins of MBP‐GhRAF36 (3 μg) and GST‐GhABI1 (3 μg) were incubated with a gradient concentration of His‐GhTOPP4aD (2–4 μg) for kinase assay at 30 °C for 30 min and separated by 12.5% SDS‐PAGE, respectively. (e) The effects of GhRAF36 and GhTOPP4aD on phosphatase activity of GhABI1 are indicated by the kinetic‐dependent curve. GhABI1 phosphatase activity of indicated concentrations was measured. The data are shown as means ± SD from three independent repeats (*n* = 3; **P < 0.01, Student's *t*‐test). (f) The effects of GhRAF36 and GhTOPP4aD on the phosphatase activity of GhABI1. The data are shown as means ± SD from three independent repeats (*n* = 3; **P < 0.01, Student's *t*‐test). (g, h) The effects of GhTOPP4aD on the phosphorylation of GhABI1 without or with salt‐stress treatment. The total proteins were extracted as kinases from 14‐day‐old VIGS or transgenic material leaves with or without 300 mM NaCl treatment. GhABI1 as a substrate was incubated with total proteins in kinase reaction buffer for 30 min at 30 °C and then separated by SDS‐PAGE. The experiments were performed three times (a–h) with similar results.

Next, we detect whether GhTOPP4aD could regulate the inhibitory effect of GhRAF36 on GhABI1 phosphatase activity. Through previous Y2H screening, we noticed that GhABI1 was also a candidate protein interacting with GhTOPP4aD (Table S1). We further determined the interaction between GhTOPP4aD and GhABI1 using point‐to‐point Y2H, BiFC and LCI assays (Figure 6a and S4a,b). Meanwhile, GST‐GhABI1 protein could directly interact with His‐GhTOPP4aD protein *in vitro* (Figure 6b). It suggests that besides GhRAF36, phosphorylated GhABI1 mediated by GhRAF36 may also be a dephosphorylation substrate of GhTOPP4aD. To test this hypothesis, we incubated GhABI1, which had been phosphorylated by GhRAF36, with different dosages of GhTOPP4aD and found that GhTOPP4aD compromised the phosphorylation of GhABI1 by GhRAF36 (Figure 6c). We further phosphorylated GhABI1‐beads with GhRAF36 to perform the dephosphorylation assay after removing GhRAF36 kinase. The results showed that GhTOPP4aD could directly dephosphorylate GhABI1 in a dosage‐dependent manner *in vitro* (Figure 6d). Subsequently, we examined the phosphatase activity of GhABI1 with or without GhTOPP4aD and found that the inhibitory effect of GhRAF36 on GhABI1 phosphatase activity could be offset by GhTOPP4aD (Figure 6e,f). Additionally, we investigated the phosphorylation status of GhABI1 in VIGS‐*GhTOPP4aD* and OE‐*GhTOPP4aD* plants. The results showed that silencing *GhTOPP4aD* further enhanced salt‐induced GhABI1 phosphorylation (Figure 6g), while overexpressing *GhTOPP4aD* exhibited the opposite result (Figure 6h). These data suggest that GhTOPP4aD enhances the phosphatase activity of GhABI1 by both inhibiting the kinase activity of GhRAF36 and directly dephosphorylating GhABI1 that is phosphorylated by GhRAF36.

### GhTOPP4aD negatively orchestrates ABA signal transduction

**Figure 7:**
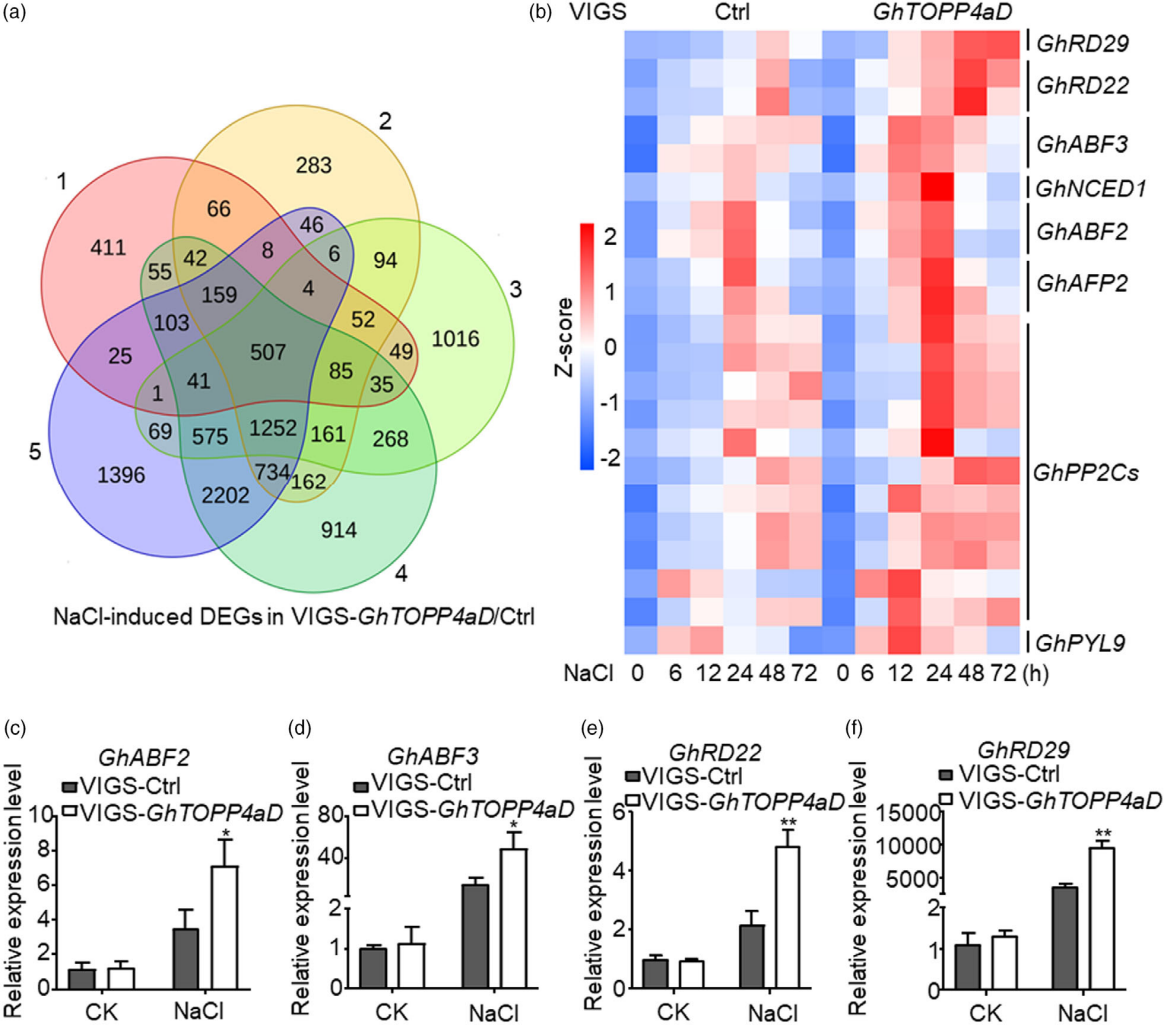
RNA‐seq analysis shows the transcriptome changes between VIGS‐*GhTOPP4aD* and VIGS‐Ctrl in response to salt stress. (a) The Venn diagram represents the number of genes that are significantly upregulated in VIGS‐*GhTOPP4aD* plants compared to VIGS‐Ctrl after NaCl treatment (*P*‐value <0.05 and log2 (Fold Change) >1). 1, S(Salt)_6h_TOPP4aD/TOPP4aD_0h VS S(Salt)_6h_ Ctrl/Ctrl _0h; 2, S(Salt)_12h_TOPP4aD/TOPP4aD_0h VS S(Salt)_12h_ Ctrl/Ctrl _0h; 3, S(Salt)_24h_TOPP4aD/TOPP4aD_0h VS S(Salt)_24h_ Ctrl/Ctrl _0h; 4, S(Salt)_48h_TOPP4aD/TOPP4aD_0h VS S(Salt)_48h_ Ctrl/Ctrl _0h; 5, S(Salt)_72h_TOPP4aD/TOPP4aD_0h VS S(Salt)_72h_ Ctrl/Ctrl _0h; (b) The heatmap displays the expression of ABA‐responsive genes upregulated in VIGS‐*GhTOPP4aD* plants in response to salt stress. (c–f) Detect the expression of ABA‐responsive genes that are upregulated in VIGS‐*GhTOPP4aD* plants in response to salt‐stress treatment. The leaf samples from VIGS‐Ctrl and VIGS‐*GhTOPP4aD* cotton seedlings were collected to detect the expression of ABA‐responsive genes with NaCl treatment by RT‐qPCR. GhActin9 was used as the internal control. The data are shown as means ± SD from three independent repeats (*n* = 3; **P < 0.01, Student's *t*‐test).

We further performed the RNA‐seq analysis with VIGS‐Ctrl or VIGS*‐GhTOPP4aD* leaf samples with or without NaCl treatment. Principal component analysis (PCA) showed significant differences within the treatments and VIGS samples, with a strong correlation of the three biological replicates (Figure S5a). There were 507 NaCl‐induced differentially expressed genes (DEGs) in VIGS plants among all the time points (Figure 7a). Gene ontology analysis revealed that the DEGs were partially enriched in biological processes such as cold response, gibberellin signalling pathway and abscisic acid signalling pathway (Figure S5b; Table S4). Interestingly, several ABA‐responsive genes, including Responsive to Desiccation 29 (*GhRD29*, Gh_A12G2329), Responsive to Desiccation 22 (*GhRD22*, Gh_A05G0390), Abscisic acid responsive elements‐binding Factor 2 (*GhABF2*, Gh_A05G2234) and Abscisic acid responsive elements‐binding Factor 3 (*GhABF3*, Gh_D12G0214), were also identified (Figure 7b). Consistent with the RNA‐seq data, quantitative reverse transcription‐polymerase chain reaction (qRT‐PCR) analysis also indicated that endogenous transcriptional expression of *GhRD29*, *GhRD22*, *GhABF2* and *GhABF3*, which are positive players in the ABA signal pathway, were elevated in *GhTOPP4aD*‐silenced plants (Figure 7c–f). These results indicate that GhTOPP4aD is a negative regulator in response to ABA.

### GhTOPP4aD‐GhRAF36‐GhABI1 module negatively regulates ABA response in cotton

**Figure 8:**
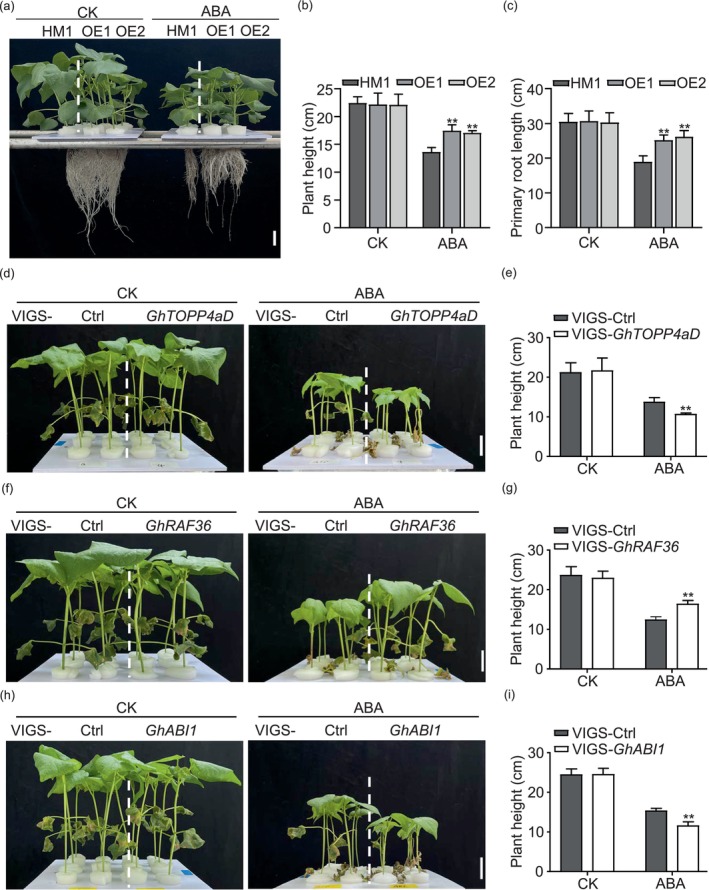
ABA‐related phenotypes in OE/VIGS‐*GhTOPP4aD*, VIGS‐*GhRAF36* and VIGS‐*GhABI1* plants. (a) Phenotypes of 14‐day‐old OE‐*GhTOPP4aD* plants after 7 days of 20 μM ABA treatment. Bar = 3 cm. (b, c) The plant height and primary root length of HM1 and OE‐*GhTOPP4aD* plants without or with ABA treatment. The data are shown as means ± SD from three independent repeats (*n* = 3; **P < 0.01, Student's *t*‐test). (d) Phenotypes of 14‐day‐old VIGS‐*GhTOPP4aD* after 7 days of 20 μM ABA treatment. Bar = 3 cm. (e) The plant height of VIGS‐Ctrl and VIGS‐*GhTOPP4aD* plants without or with ABA treatment. The data are shown as means ± SD from three independent repeats (*n* = 3; **P < 0.01, Student's *t*‐test). (f) Phenotypes of 14‐day‐old VIGS‐*GhRAF36* plants after 7 days of 20 μM ABA treatment. Bar = 3 cm. (g) The plant height of VIGS‐Ctrl and VIGS‐*GhRAF36* plants without or with ABA treatment. The data are shown as means ± SD from three independent repeats (*n* = 3; **P < 0.01, Student's *t*‐test). (h) Phenotypes of 14‐day‐old VIGS‐*GhABI1* plants after 7 days of 20 μM ABA treatment. Bar = 3 cm. (i)The plant height of VIGS‐Ctrl and VIGS‐*ABI1* plants without or with ABA treatment. The data are shown as means ± SD from three independent repeats (*n* = 3; **P < 0.01, Student's *t*‐test).

Next, we analysed the ABA‐induced growth inhibition phenotype in OE‐*GhTOPP4aD* and VIGS‐*GhTOPP4aD* plants and found that *GhTOPP4aD‐*overexpressed plants showed insensitivity to ABA‐induced growth inhibition compared to the recipient plants (Figure 8a), displaying a more vigorous growth in terms of leaf size, plant height and primary root length (Figures 8b,c and S6a). However, *GhTOPP4aD*‐silenced plants exhibited significant growth inhibition of leaf size and plant height induced by ABA compared to the recipient plants (Figures 8d,e, and S6b). Meanwhile, we found that VIGS‐*GhRAF36* plants were more insensitive to and VIGS‐*GhABI1* plants were more sensitive to ABA‐induced growth inhibition of leaf size and plant height (Figures 8f–i and S6c,d). We also examined the kinase activity of GhRAF36 and the phosphorylation of GhABI1 in VIGS‐*GhTOPP4aD* or VIGS‐*GhRAF36* plants and VIGS‐Ctrl plants with or without ABA treatment. The results showed that ABA could induce the phosphorylation of GhRAF36 and GhABI1 in VIGS‐Ctrl plants (Figure S6e,f). Moreover, silencing *GhTOPP4aD* further enhanced GhRAF36 phosphorylation (Figure S6e), and silencing *GhRAF36* significantly inhibited GhABI1 phosphorylation compared with that in VIGS‐Ctrl plants (Figure S6f), suggesting that salt probably induces the production of ABA, which in turn activates GhRAF36 and subsequently phosphorylates and inhibits GhABI1. In summary, these data indicate that GhRAF36 is a positive modulator in response to ABA, while GhTOPP4aD and GhABI1 are negative players.

**Figure 9:**
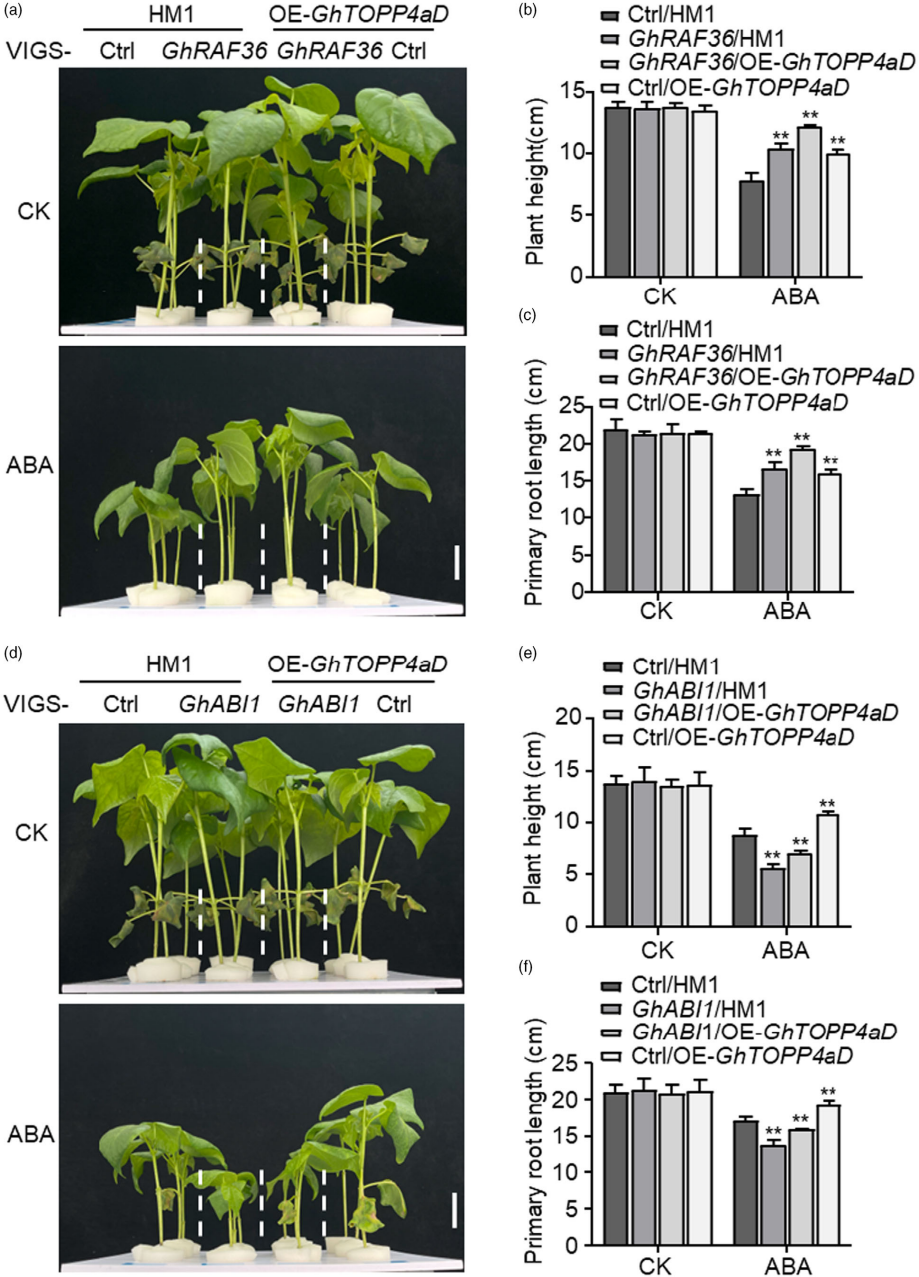
The effect of silencing *GhABI1* and *GhRAF36* on the ABA response of OE‐*GhTOPP4aD* plants. (a) Silencing *GhRAF36* enhances the ABA insensitivity of OE‐*GhTOPP4aD* plants under 20 μM ABA treatment. Bar = 3 cm. (b, c) Determination of plant height and primary root length in (a). The data are shown as means ± SD from three independent repeats (*n* = 3; **P < 0.01, Student's *t*‐test). (d) Silencing *GhABI1* compromises the ABA insensitivity of OE‐*GhTOPP4aD* plants under 20 μM ABA treatment. Bar = 3 cm. (e, f) Determination of plant height and primary root length in (d). The data are shown as means ± SD from three independent repeats (*n* = 3; **P < 0.01, Student's *t*‐test).

**Figure 10:**
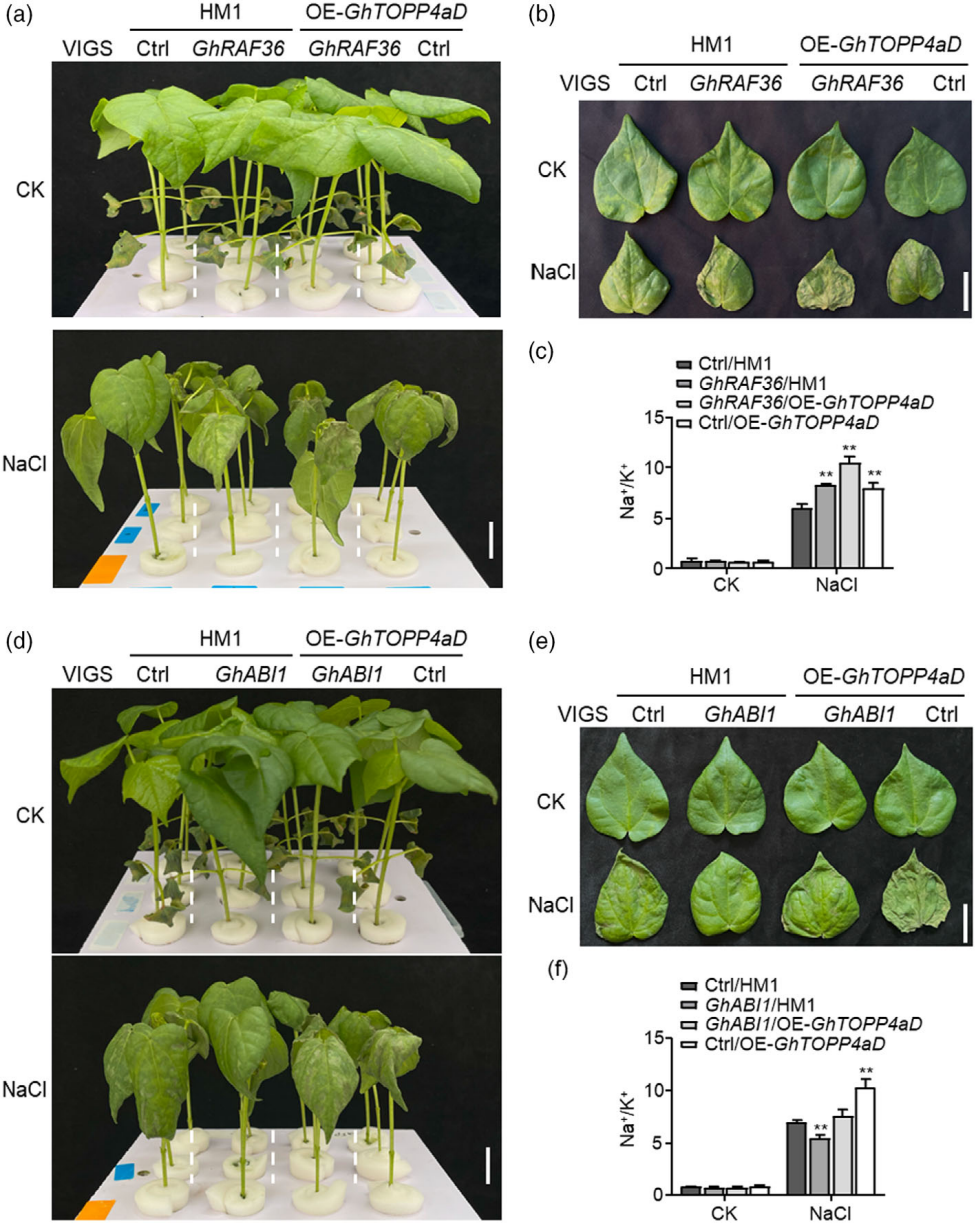
Effect of silencing *GhABI1* or *GhRAF36* on the salt response of OE‐*GhTOPP4aD* plants. (a, b) Silencing *GhRAF36* enhances the salt sensitivity of OE‐*GhTOPP4aD* plants under NaCl treatment. The second leaf was collected for the photograph. Bar = 3 cm. (c) Na^+^/K^+^ in VIGS‐Ctrl/HM1, VIGS‐*GhRAF36*/HM1, VIGS‐*GhRAF36*/OE‐*GhTOPP4aD* and VIGS‐Ctrl/OE‐*GhTOPP4aD* without or with NaCl treatment. (d, e) Silencing *GhABI1* reduces the salt sensitivity of OE‐*GhTOPP4aD* plants under NaCl treatment. The second leaf was collected for the photograph. Bar = 3 cm. (f) Na^+^/K^+^ in VIGS‐Ctrl/HM1, VIGS‐*GhRAF36*/HM1, VIGS‐*GhRAF36*/OE‐*GhTOPP4aD* and VIGS‐Ctrl/OE‐*GhTOPP4aD* without or with NaCl treatment. The data are shown as means ± SD from three independent repeats (*n* = 3; **P < 0.01, Student's *t*‐test). The experiments were performed three times with similar results.

To explore the epistatic effects of *GhTOPP4aD*, *GhRAF36* and *GhABI1* in response to ABA and salt stress, we silenced *GhRAF36* or *GhABI1* in OE‐*GhTOPP4aD* and its recipient plants to perform the growth inhibition assay. The data showed that both *GhRAF36‐*silenced plants and *GhTOPP4aD‐*overexpressed plants exhibited significant insensitivity to ABA‐induced growth inhibition of plant height compared to the control plants, which was further enhanced when silencing *GhRAF36* in OE‐*GhTOPP4aD* plants, indicating that *GhTOPP4aD* and *GhRAF36* synergistically regulate the inhibitory effect of ABA on cotton growth (Figures 9a–c and S7a,b). Meanwhile, we observed that silencing *GhABI1* in overexpression of *GhTOPP4aD* plants significantly compromised the insensitivity to ABA‐induced growth inhibition in OE‐*GhTOPP4aD* plants, suggesting that *GhABI1* possesses an epistatic regulatory role relative to *GhTOPP4aD* in response to ABA‐mediated growth inhibition (Figures 9d–f and S7c,d). In addition, silencing *GhRAF36* further enhanced salt sensitivity of OE‐*GhTOPP4aD* plants with lower fresh weight, reduced chlorophyll content and a higher Na^+^/K^+^ (Figures 10a–c and S8a,b), while silencing *GhABI1* compromised salt sensitivity of OE‐*GhTOPP4aD* plants with higher fresh weight and chlorophyll content, along with a lower Na^+^/K^+^ (Figures 10d–f and S8c,d), indicating that *GhRAF36* or *GhABI1* possesses an epistatic regulatory role relative to GhTOPP4aD in response to salt stress. Together, these results indicate that the GhTOPP4aD‐GhRAF36‐GhABI1 module plays a key role in ABA and salt‐stress response.

## Discussion

**Figure 11:**
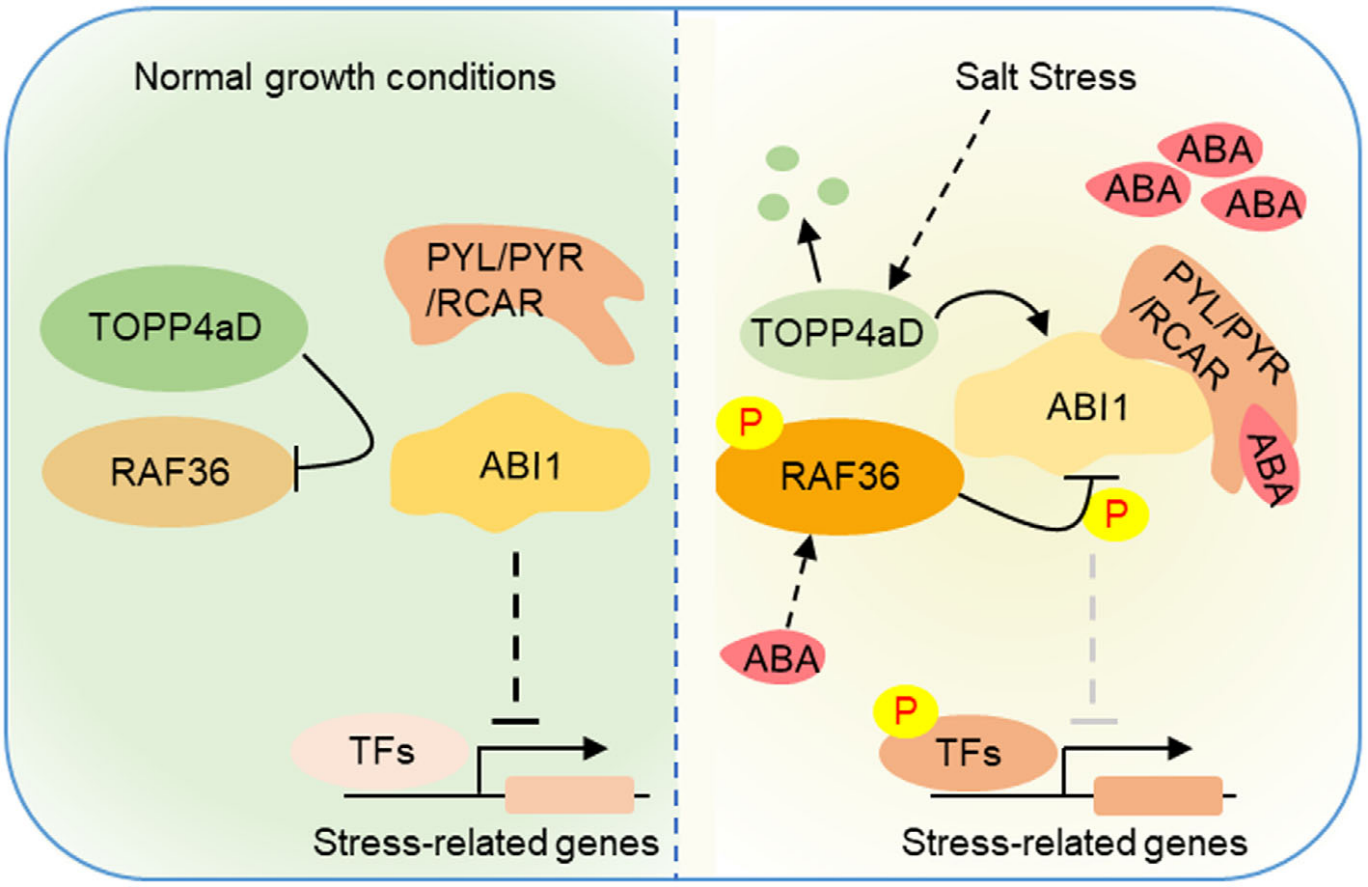
A proposed working model for the role of GhTOPP4aD‐GhRAF36‐GhABI1 in response to salt stress. GhTOPP4aD inhibits ABA signalling through physical interactions with GhRAF36 to inhibit its kinase activity, which enhances the phosphatase activity of GhABI1. Salt stress induces the degradation of GhTOPP4aD, allowing the activated GhRAF36 to phosphorylate GhABI1 and inhibit its phosphatase activity, thereby enhancing ABA signalling and salt tolerance in cotton.

Despite existing research on cotton salt tolerance, the complex mechanism of salt‐stress responses mediated by reversible phosphorylation modification of the stress‐related proteins remains to be fully explored. In this study, our results demonstrate that GhRAF36 and GhTOPP4aD reversibly regulate the phosphorylation and phosphatase activity of GhABI1 in response to ABA and salt stress in cotton. Specifically, under normal conditions, GhTOPP4aD binds to GhRAF36 and GhABI1 to inhibit the phosphorylation and enhance the phosphatase activity of GhABI1 mediated by GhRAF36, thus inhibiting SnRK2s kinase activity and negatively regulating the ABA signalling pathway, allowing cotton to undergo normal growth and development. Salt stress promotes the degradation of GhTOPP4aD protein to release GhRAF36 kinase activity and induce the phosphorylation of GhABI1, leading to a weaker phosphatase activity of GhABI1 and activated ABA signalling pathway (Figure 11). We suggest that GhTOPP4aD may act as a switch to regulate the balance between cotton growth and salt‐stress response.

### GhRAF36 phosphorylates GhABI1 to inhibit its phosphatase activity

Protein phosphatases ABI1/2 are key negative component regulators in the ABA signalling pathway. Similarly, we also notice that GhABI1 negatively regulates cotton's response to ABA and salt stress (Figures 4g–k and 8h–i). When PYR/PYL/RCAR receptors perceive ABA, the phosphatase activity of AtABI1 will be inhibited, and the degradation of AtABI1 is mediated by plant U‐box type E3 ligases PUB12/13, and the UBC27–AIRP3 ubiquitination complex promotes its degradation (Kong *et al*., 2015; Pan *et al*., 2020). Recent studies have shown that kinase‐mediated phosphorylation at different residues can alter the phosphatase activity of PP2Cs. AtTMK4 enhances AtABI2 activity by phosphorylating its residues S139, S140 and S266. The phosphorylation‐mimic AtABI2^S139DS140DS266D^ can complement the ABA hypersensitive phenotype of the loss‐of‐function mutant *Atabi1‐2abi2‐2*, while the non‐phosphorylated form AtABI2^S139AS140AS266A^ cannot (Li *et al*., 2021a). Conversely, AtTMK1 inhibits the phosphatase activity of AtABI2 by phosphorylating residue T321. The non‐phosphorylated form ABI2^T321A^, but not the phosphorylation‐mimic form ABI2^T321D^, could rescue the hypersensitive ABA responses in the *Atabi1‐2abi2‐2* mutant (Yang *et al*., 2021). Here, we found that GhRAF36 inhibits GhABI1 activity in cotton by directly phosphorylating its T124 and S357 residues, which are unique in cotton (Figures 4a–e and S3b–d). Mutating either of them to alanine (A) can affect the phosphorylation of GhABI1 mediated by GhRAF36 (Figure 4e). Studies have shown that the regulatory mechanisms of the ABA signalling pathway in plants undergo adaptive changes in response to different environmental stresses (Cutler *et al*., 2010). In Arabidopsis, AtRAF22 enhances AtABI1 phosphatase activity to negatively regulate ABA signalling in response to drought stress (Sun *et al*., 2022). Here, we found that cotton GhRAF36 inhibits GhABI1 phosphatase activity to activate the ABA signalling pathway under salt stress. Furthermore, GhRAF36 is a paralogue of AtRAF36 and exhibits evolutionary differences with AtRAF22 in their overall protein sequences and kinase domain sequences. It suggests that diverse stress signals may inhibit or activate ABA signalling by activating different members of the RAF family. In addition, different phosphorylation sites on ABI1/2 can enhance or inhibit the phosphatase activity through various modifications to the protein structure (Nolen *et al*., 2004; Ubersax and Ferrell Jr., 2007). Arabidopsis AtRAF22 phosphorylates AtABI1 on T331 and S416 residues (Sun *et al*., 2022). However, cotton GhRAF36 directly phosphorylates GhABI1 on unique T124 and S357 residues, which are not conserved in the AtABI1 protein sequence (Figures 4a–e and S3a–c), indicating that the phosphorylation sites on GhABI1 may obscure the phosphatase catalytic active site of ABI through changes in the protein structure. Together, cotton has undergone polyploidization events, and GhRAF36, as a RAF family member, has acquired inhibitory regulatory capabilities to ABI1 phosphatase activity through subfunctionalization or neofunctionalization.

### GhRAF36 positively regulates ABA and salt‐stress responses in cotton

Previous studies have suggested that the B2, B3 and B4 subfamilies of RAFs are upstream kinases that phosphorylate and activate SnRK2s, playing a crucial role in mediating osmotic stress and ABA responses (Lin *et al*., 2020; Soma *et al*., 2023). Recently, two members of the B1 subfamily, AtRAF13 and AtRAF15, are also characterized to act as upstream kinases to phosphorylate and activate SnRK2s, as ABA‐triggered SnRK2.6 activation and stomatal closure are impaired in *Atraf15* mutants (Wang *et al*., 2023). Moreover, two members of the C subgroup, AtRAF22 and AtRAF36, are direct substrates of SnRK2s and negatively regulate ABA signalling (Kamiyama *et al*., 2021; Sun *et al*., 2022). Here, we clarified that VIGS‐*GhRAF36* plants are insensitive to ABA but sensitive to salt stress (Figures 3g–i and 8f–g), suggesting that it is a positive regulator in ABA and salt‐stress responses. It is likely achieved through the phosphorylation sites of GhABI1 regulated by GhRAF36. We have also noticed that the phosphorylation of GhRAF36 is induced by salt treatment and is further enhanced when silencing *GhTOPP4aD*, indicating that there is probably a kinase that counteracts with GhTOPP4aD to regulate the kinase activity of GhRAF36.

### GhTOPP4aD negatively modulates ABA signalling and salt tolerance through the counteraction with GhRAF36 in cotton

GhTOPP4aD belongs to the Protein Phosphatase 1 (PP1) family, which is a class of serine/threonine protein phosphatases that contain catalytic and regulatory subunits. Previous research has shown that the members of the PP1 family can participate in plant stress response. In *Arabidopsis thaliana*, AtTOPP1 interacts with its regulatory factor At Inhibitor‐2 (AtI‐2) to negatively regulate the ABA signalling pathway. Both *Attopp1* and *Atati‐2* mutants are sensitive to ABA and salt stress (Hou *et al*., 2016). PP1 Regulatory Subunit 3 (PP1R3) forms a holoenzyme with TOPPs to mediate ABA signal transduction, and *Atpp1r3* mutants are also sensitive to salt stress (Zhang *et al*., 2020). In rice, overexpression of *OsPP1a* enhances rice salt tolerance (Liao *et al*., 2016). Additionally, overexpression of *GmTOPP13* enhances drought tolerance in tobacco (Wang *et al*., 2021). Here, we have shown that GhTOPP4aD dephosphorylates GhABI1 regulated by GhRAF36 and negatively participates in cotton salt stress and ABA response (Figures 1, 6 and 8, 9, 10). VIGS‐*GhTOPP4aD* plants show significantly enhanced salt tolerance and are sensitive to ABA compared to that in VIGS‐Ctrl plants (Figures 1a–f and 8d). On the contrary, overexpression of *GhTOPP4aD* enhances salt‐stress sensitivity but reduces ABA sensitivity compared to recipient plants (Figures 1h–l and 8a–c). Importantly, silencing *GhRAF36* further enhances ABA insensitivity in OE‐*GhTOPP4aD* plants, while silencing *GhABI1* compromises it (Figure 9). Together, the data indicate that GhRAF36 works together with GhTOPP4aD to regulate GhABI1 and ABA response in cotton. Consistently, our RNA‐seq data elucidates that GhTOPP4aD could orchestrate the expression of a large number of ABA‐related genes (Figure 7b). Under salt stress, the activation of ABA signal may be due to the protein degradation of endogenous GhTOPP4aD protein (Figure 2a,b). Thus, it is worthwhile to further identify the regulators that mediate the degradation of GhTOPP4aD. At the later stages of stress, ABA signalling gradually weakens (Li *et al*., 2021a; Sun *et al*., 2022; Wang *et al*., 2018). Whether the activity of GhRAF36, as a positive regulator in the ABA response, is negatively regulated by other TOPPs through dephosphorylation or SnRK2s through phosphorylation, thus maintaining a dynamic balance between plant growth and stress adaptation, remains to be further explored.

Numerous studies have shown that salt stress could induce ABA accumulation to mediate salt‐stress responses (Chen *et al*., 2021; Zhu, 2016). In rice, salt stress upregulates the expression level of cell wall cellulose synthase‐like D4 protein (*OsCSLD4*), and overexpression of *OsCSLD4* enhances the expression of ABA biosynthetic genes to increase ABA content and improve salt tolerance (Zhao *et al*., 2022). In addition, under salt stress, the SnRK2‐type protein kinase SAPK9 in rice phosphorylates MADS‐box transcription factor 23 (OsMADS23) and activates its transcriptional activity towards *OsNCED2*, *OsNCED3* and *OsNCED4*, which encode key enzymes catalysing ABA synthesis, thereby promoting the biosynthesis of endogenous ABA and positively regulating salt tolerance in rice (Li *et al*., 2021b). However, it remains unclear how salt stress triggers the ABA signalling pathway, especially in cotton. Our study found that GhTOPP4aD exhibited a negative regulatory effect on cotton's response to ABA and salt stress (Figures 1 and 8), and salt stress induced the degradation of GhTOPP4aD, which could inhibit gene expression related to the ABA signalling pathway (Figures 2 and 7), indicating that GhTOPP4aD may be a switch to modulate salt‐triggered ABA signals in cotton. Moreover, the kinase activity of GhRAF36, a dephosphorylation substrate of GhTOPP4aD, was induced by both ABA and salt stress (Figures 3 and S6). Similarly, the phosphorylation of GhABI1, a substrate of GhRAF36, was also induced by ABA and salt stress (Figures 4 and S6). Consistently, GhRAF36 and GhABI1 exhibited an epistatic regulatory role relative to GhTOPP4aD in response to both ABA and salt stress (Figures 9 and 10). Taken together, we provided a novel insight that the GhTOPP4aD‐GhRAF36‐GhABI1 module regulates salt tolerance through the ABA‐dependent signalling pathway in cotton.

## Materials and methods

### Plant materials and growth conditions

*Gossypium hirsutum* (L.) XinShi 17 was preserved in our laboratory. Cotton transgenic recipient plants HM 1 and OE‐*GhTOPP4aD* plants were provided by Wuhan Towin Biotechnology Company Limited in China. Cotton seeds were germinated in sand and then transplanted into Hoagland nutrient solution five days (d) after sowing (DAS). OE‐*GhTOPP4aD* and seedlings prepared for injection were grown at 24 °C, 60% relative humidity and 400 μmol m^−2^ s^−1^ light with a 14‐h (h) light: 10‐h dark photoperiod. The *Nicotiana benthamiana* plants were grown in pots with soil: vermiculite (w/w), 1:1 in a controlled growth chamber at 22 °C, 60% relative humidity, and 80 μmol m^−2^ s^−1^ light with a 14‐h light: 10‐h dark photoperiod.

### Virus‐induced gene silencing (VIGS) assay

The VIGS assay was performed as previously described (Mu *et al*., 2019). In brief, the plasmids of *pTRV‐RNA1* or *pTRV*‐*RNA2* (Ctrl, *GhCLA1*, *GhTOPP4aD*, *GhRAF36* and *GhABI1*) were transferred into *Agrobacterium tumefaciens* strain GV3101. *Agrobacterial* culture was grown overnight at 28 °C in YEP liquid medium (50 μg/mL kanamycin, 25 μg/mL gentamicin, 10 mM MES and 20 μM acetosyringone). The cells were pelleted by centrifugation at 8000 rpm at room temperature (RT) for 5 min (min), and resuspended in infiltration buffer (10 mM MgCl_2_, 10 mM MES and 200 mΜ acetosyringone). The *pTRV‐RNA2* at OD_600_ 1.5 was mixed with *pTRV‐RNA1* at a 1:1 ratio and infiltrated into two fully expanded cotyledons of three‐day‐old plants grown in a hydroponic system using a needle‐less syringe. The VIGS‐GhCLA1 plants were used as a marker to monitor silencing reliability. VIGS experiments were repeated at least three times with more than six plants for each construct per repeat.

### qRT‐PCR

The total RNA of VIGS plants was extracted with the Plant RNA Mini Kit (Aidlab, Beijing); 2 μg RNA was used for first‐strand cDNA synthesis with the first‐strand cDNA synthesis kit (Aidlab, Beijing). The synthesized cDNA was used for qRT‐PCR with iTaq SYBR green Supermix (Bio‐Rad) in an Applied Biosystems 7500 Fast real‐time PCR machine following the standard protocol. GhActin9 was used as the internal control. The primers for qRT‐PCR are listed in Table S3.

### Determination of sodium and potassium ion content

Grind the dried plant samples into a fine dry powder, accurately weigh out 0.2 g and place it in a 10 mL centrifuge tube. Add 5 mL of 1 mol/L HCl for extraction, and then place the tube in a shaker at 28 °C for overnight oscillation. After that, filter the extract through a 9 cm qualitative filter paper. Dilute the leaching solution with HCl to 5 mL and measure absorbance with flame spectrophotometer readings.

### Determination of chlorophyll content

About 0.2 g leaf from 14‐day‐old plants was placed in 80% acetone for efficient pigment leaching, followed by its spectrophotometric analysis at 663 nm and 645 nm.

### Subcellular localization of GhTOPP4aD

The CDS of *GhTOPP4aD* was amplified without a stop codon and inserted into the pHBT‐GFP vector. The fusion construct (*35S*::GhTOPP4aD‐GFP) and the control vector (*35S*::GFP) were introduced into *Agrobacterium tumefaciens* strain GV3101 for transient expression in cotton protoplasts. Fluorescence signals in cotton protoplasts were examined 12 h after transfection using the Zeiss LSM900 confocal laser scanning microscope (Carl Zeiss AG, Oberkochen, Germany). NLS‐RFP and Pm‐RFP were used as a nuclear marker and a plasma membrane marker, respectively. Chl represents the detected autofluorescence chloroplast signal. The GFP fluorescence was excited at 488 nm and emissions were detected between 490 and 530 nm. The RFP fluorescence was excited at 587 nm and emissions were detected between 590 nm and 620 nm. Chloroplast autofluorescence was excited at 488 nm and emissions were detected between 640 and 700 nm. Images were captured in multichannel mode with a bright field and processed with Zeiss ZEN microscope software.

### Screen of Y2H cDNA library

The full‐length sequence of *GhTOPP4aD* was amplified and inserted into the pGBKT7 vector, then transformed into Y2H‐Gold yeast cells with the cotton cDNAs. The transformed cells were plated onto agar plates containing synthetic dropout SD/‐Trp‐Leu‐His supplemented with 10 mmol/L 3‐amino‐1,2,4‐triazole (3‐AT) for selecting the positive colonies. A single colony was picked up to perform PCR amplification and sent to sequencing.

### Yeast two‐hybrid assay

The full‐length sequences of *GhTOPP4aD*, *GhRAF36* and *GhABI1* were amplified and inserted into the pGBKT7 or pGADT7 vector. The different plasmid pairs were co‐transformed into Y2H‐Gold yeast cells. The transformation was dilution plated onto agar plates containing synthetic dropout SD/‐Trp‐Leu and synthetic dropout SD/‐Trp‐Leu‐His supplemented with 10 mmol/L 3‐amino‐1,2,4‐triazole (3‐AT) or SD/‐Trp‐Leu‐His‐Ade medium for 4–5 days to test the interaction. The primer sequences used for cloning were listed in Table S3.

### Recombinant protein expression and purification

The CDS of corresponding genes was cloned into pET28a (His tag), pGEX4T‐1 (GST tag and pMAL‐c2X (MBP tag)), respectively. These vectors were transformed into *E. coli* BL21. The recombinant proteins with different tags were induced with 0.2 mM IPTG for 14–16 h at 18 °C. The culture was collected by centrifugation at 6000 g for 3 min at 4 °C. The recombinant proteins were purified using Ni Sepharose resin (17 371 202, GE Healthcare) for His tag, with Amylose Resin High Flow (BioLabs, #E8022) for MBP tag, or Pierce™ Glutathione Agarose resin (Thermo Fisher Scientific, Waltham, MA, USA) for GST tag, respectively.

### Measurements of phosphatase activity

GhTOPP4aD phosphatase activity was assessed by incubating recombinant GST‐GhTOPP4aD protein with 1 mM Pyronitrophenyl phosphate (pNPP) as the substrate in the phosphatase buffer (50 mM Tris‐HCl, pH 7.0, and 2 mM DTT). The absorbance was measured at 405 nm.

PP2C phosphatase activity was measured using a Ser/Thr Phosphatase Assay Kit (Promega; V2460) as described (Park *et al*., 2009). In brief, the corresponding CDS of *GhTOPP4aD*, *GhABI1*, *GhABI1* ^*T124A*^, *GhABI1* ^*S357A*^, *GhABI1* ^*mut2A*^ and *GhABI1* ^*mut2D*^ were fused with the pET‐28a vectors and transformed into the *E. coli* strain BL21. The recombinant proteins were purified on Ni Sepharose and eluted with elution buffer (50 mM Tris‐HCl, pH 7.2, and 400 mM imidazole). None of the buffers used for protein purification contained phosphate. To measure phosphatase activity, different combinations of GhTOPP4aD and GhABI1 proteins were mixed with 5 mL of 1 mM phosphopeptide [RRA(phosphoT)VA] and 10 μL of PP2C 5 × reaction buffer (250 mM imidazole, pH 7.2, 1 mM EGTA, 25 mM Mg_2_Cl, 0.1% β‐mercaptoethanol, and 0.5 mg/mL BSA) at 25 °C for 30 min. The reactions were stopped by adding 50 μL of protease solution, and the plate was incubated at room temperature for 30 min. The absorbance was measured at 630 nm in a Microplate Reader (EnSpire 2300).

### Protein degradation assay

Fourteen‐day‐old OE‐*GhTOPP4aD* seedlings were treated with 150 μM protein biosynthesis inhibitor cycloheximide (CHX) or 150 μM CHX plus 300 mM NaCl for the indicated time. Seedlings were collected and immediately frozen in liquid nitrogen. Proteins were extracted with extraction buffer (25 mM Tris‐HCl, pH 7.5, 10 mM NaCl, 10 mM MgCl_2_, 0.5% Tween 20, 1 mM EDTA, 1 mM DTT, 4 mM PMSF), and the abundance of GhTOPP4aD was determined using the anti‐GhTOPP4aD antibody (Provided by Nanjing GenScript Biotech Corporation) by western blotting analysis.

### LCI assay

The LCI assays were performed as reported (Chen *et al*., 2008). Briefly, the CDS of *GhTOPP4aD*, *GhABI1* and *GhRAF36* was cloned into *pCAMBIA1300‐nLUC/pCAMBIA1300‐cLUC*. The constructs were co‐transformed into *Agrobacterium* GV3101 and co‐injected into *N. benthamiana* leaves for 48–72 h. The luciferase (LUC) signal was captured by a cold charge‐coupled device camera. The primers used for the LCI assay are listed in Table S3.

### Pull‐down assay

Purified GST‐GhABI1 or MBP‐GhRAF36 proteins (6 μg) coupled with resin were added to pull‐down buffer (50 mmol/L Tris‐HCl, 150 mmol/L NaCl and 10 mmol/L MgCl_2_, pH 8.0) at 4 °C. After brief centrifugation at 100 × *g* for 5 min at 4 °C, the supernatant was removed. About 3 μg of the His‐GhTOPP4aD protein was added to the resin, along with pull‐down buffer, and rotated at 4 °C for 12 h for protein binding. Then the resin was washed six times with pull‐down buffer to remove the nonspecifically bound proteins, combined with 80 μL of pull‐down buffer and 20 μL of 5 × sodium dodecyl sulphate (SDS) loading buffer, and boiled for 10 min. After centrifugation at 12 000 × *g* for 1 min, the supernatant was subjected to immunoblot analysis. Proteins that were pulled down were analysed by western blotting using anti‐GST, anti‐MBP, or anti‐His antibodies.

### BiFC assay

Constructs expressing GhTOPP4aD‐nYFP + GhRAF36‐cYFP, GhRAF36‐nYFP + GhABI1‐cYFP, GhTOPP4aD‐nYFP + GhABI1‐cYFP were transformed into *N. benthamiana* leaves and expressed for 48 h. Subsequently, the signal was visualized using confocal microscopy.

### *In vitro* dephosphorylation assay

The purified recombinant proteins of MBP‐GhRAF36 (2 μg) were incubated with His‐GhTOPP4aD (4 μg) or CIPA at 30 °C for 30 min in phosphatase buffer (25 mM Tris‐HCl, pH 7.4, 12 mM MgCl_2_ and 2 mM DTT). After the reaction, 5 × SDS sample buffer was added to the reaction mixture and boiled for 10 min, and the samples were separated by 12.5% SDS‐PAGE. The phosphorylation of the MBP‐GhRAF36 was detected using a pIMAGO‐biotin phosphoprotein detection kit (Iliuk *et al*., 2011; Iliuk and Tao, 2015).

### *In vitro* kinase assay

The purified recombinant proteins of GST‐GhABI1 (2 μg) and MBP‐GhRAF36 (2 μg) were incubated at 30 °C for 30 min in kinase reaction buffer (25 mM Tris‐HCl, pH 7.4, 5 mM MgCl_2_, 50 μM ATP, 1 mM DTT). After the reaction, 5 × SDS sample buffer was added to the reaction mixture and boiled for 10 min; the samples were separated by 10% SDS‐PAGE. The phosphorylation of GST‐GhABI1 was detected using a pIMAGO‐biotin phosphoprotein detection kit.

About 0.2 g of 14‐day‐old VIGS plant leaves were homogenized in 200 μL total protein extraction buffer (50 mM Tris‐HCl at pH7.4, 150 mM NaCl, 0.5 mM EDTA, 5% Glycerol, 2.5 mM MgCl_2_ and 1% Triton X‐100). The total protein of VIGS plants was used to phosphorylate recombinant GST‐GhABI1 or pre‐dephosphorylated GhRAF36 beads in 80 μL reaction buffer (25 mM Tris‐HCl, pH 7.4, 5 mM MgCl_2_, 50 μM ATP, 1 mM DTT) for 30 min at 30 °C. Phosphorylated GST‐GhABI1 or GhRAF36 beads were collected by centrifugation and washed three times with kinase buffer. The reactions were stopped by the addition of 5 × SDS loading buffer after incubation. The phosphorylation was detected using a pIMAGO‐biotin phosphoprotein detection kit.

### LC–MS/MS

To identify the putative phosphorylation sites of GhABI1 regulated by GhRAF36 *in vitro*, 8 μg purified GST‐GhABI1 and 4 μg MBP‐GhRAF36 were incubated at 30 °C for 30 min in kinase reaction buffer. The reaction products were reduced by adding DTT and alkylated with iodoacetamide, followed by digestion with trypsin at 37 °C for 12 h. The results were analysed by LC–MS/MS as described (Kong *et al*., 2015).

### Analysis of RNA‐seq data

Fourteen‐day‐old VIGS‐Ctrl and VIGS‐*GhTOPP4aD* seedlings were treated with 250 mM NaCl. Leaf samples from the VIGS plants were collected at 0, 6, 12, 24, 48 and 72 h, respectively. The total RNA was then isolated with the Plant RNA Mini Kit (Aidlab, Beijing); sequencing and analysis were carried out by Beijing Allwegene Technology. PCA, Venn diagram, heatmap and GO enrichment analysis were performed using Allwegene Cloud tools (http://218.2.224.234:8888/tools).

## Accession numbers

Cotton gene sequence information can be obtained from the CottonFGD database (https://cottonfgd.org/), accession numbers as following *GhTOPP4aA* (Gh_A10G2014), *GhTOPP4aD* (Gh_D10G2504), *GhTOPP4bA* (Gh_A13G1484), *GhTOPP4bD* (Gh_D13G1801), *GhTOPP4cA* (Gh_A05G0908), *GhTOPP4cD* (Gh_D07G0991), *GhTOPP4dA* (Gh_A03G0379), *GhTOPP4dD* (Gh_D03G1163), *GhRAF36* (Gh_D05G1535), *GhABI1* (Gh_D13G2089), *GhRD29* (Gh_A12G2329), *GhRD22* (Gh_A05G0390), *GhABF2* (Gh_A05G2234), *GhABF3* (Gh_D12G0214), *GhNCED1* (Gh_A12G2329), *GhAFP2* (Gh_D13G0860), *GhAHG1* (Gh_A12G2380), *GhPP2CA* (Gh_D13G0199), *GhAIP1* (Gh_D05G3907), *GhABI2* (Gh_A07G0123), *GhABI1* (Gh_A13G1741), *GhPYL9* (Gh_D11G1013).

## Conflict of interest

The authors declare that they have no conflict of interest.

## Author contributions

F.L. and P.C. designed the experiments. F.L. built the cotton yeast two‐hybrid libraries in P.H.'s laboratory. L.Z. detected the salt stress phenotype in VIGS‐*GhTOPP4aD* plants and performed the protein subcellular localization assays. P.C. performed the other experiments and carried out data analyses. F.L. and P.C. wrote the manuscript. Z.L., X.T. and M.D. revised the manuscript. All the authors discussed and approved the final manuscript.

## Supporting information


**Figure S1** GhTOPP4aD negatively regulates salt tolerance in cotton.
**Figure S2** Silencing of *GhRAF36* increases the Na^+^ content under salt stress.
**Figure S3** GhRAF36 phosphorylates GhABI1 at T124 and S357.
**Figure S4** GhTOPP4aD interacts with GhABI1.
**Figure S5** Transcriptome features and relationships among all samples.
**Figure S6** Analyze the leaves phenotypes of OE/VIGS‐*GhTOPP4aD*, VIGS‐*GhRAF36*, and VIGS‐*GhABI1* after ABA treatment.
**Figure S7** Effect of silencing *GhABI1* or *GhRAF36* on the ABA response of OE‐*GhTOPP4aD* plants.
**Figure S8** Effect of silencing *GhRAF36* or *GhABI1* on the salt response of OE‐*GhTOPP4aD* plants.
**Table S1** Partial candidate interaction proteins obtained by screening a yeast two‐hybrid complementary DNA (cDNA) library with GhTOPP4aD.
**Table S2** Partial candidate interaction proteins obtained by screening a yeast two‐hybrid complementary DNA (cDNA) library with GhRAF36.
**Table S3** Primers used in this study.
**Table S4** Gene ontology enrichment analysis of the differentially expressed genes (DEGs) from (Figure 7a).

**Data S1** Gene ontology enrichment analysis of the differentially expressed genes (DEGs).

## Data Availability

All data supporting the findings are contained in this manuscript. Additional Supporting Information can be found in the Supporting Information section at the end of the article.

## References

[pbi70166-bib-0001] Bartels, D. and Sunkar, R. (2005) Drought and Salt Tolerance in Plants. Crit. Rev. Plant Sci. 24, 23–58.

[pbi70166-bib-0002] Ceulemans, H. and Bollen, M. (2004) Functional diversity of protein phosphatase‐1, a cellular economizer and reset button. Physiol. Rev. 84, 1–39. 14715909 10.1152/physrev.00013.2003

[pbi70166-bib-0003] Chen, H. , Zou, Y. , Shang, Y. , Lin, H. , Wang, Y. , Cai, R. , Tang, X. *et al*. (2008) Firefly luciferase complementation imaging assay for protein‐protein interactions in plants. Plant Physiol. 146, 368–376. 18065554 10.1104/pp.107.111740 PMC2245818

[pbi70166-bib-0004] Chen, L. , Lu, B. , Liu, L. , Duan, W. , Jiang, D. , Li, J. , Zhang, K. *et al*. (2021) Melatonin promotes seed germination under salt stress by regulating ABA and GA(3) in cotton (Gossypium hirsutum L.). Plant Physiol. Biochem. 162, 506–516. 33773227 10.1016/j.plaphy.2021.03.029

[pbi70166-bib-0005] Cutler, S.R. , Rodriguez, P.L. , Finkelstein, R.R. and Abrams, S.R. (2010) Abscisic acid: emergence of a core signaling network. Annu. Rev. Plant Biol. 61, 651–679. 20192755 10.1146/annurev-arplant-042809-112122

[pbi70166-bib-0006] Dohadwala, M. , da Cruz e Silva, E.F. , Hall, F.L. , Williams, R.T. , Carbonaro‐Hall, D.A. , Nairn, A.C. , Greengard, P. *et al*. (1994) Phosphorylation and inactivation of protein phosphatase 1 by cyclin‐dependent kinases. Proc. Natl. Acad. Sci. U. S. A. 91, 6408–6412. 8022797 10.1073/pnas.91.14.6408 PMC44211

[pbi70166-bib-0007] Farkas, I. , Dombradi, V. , Miskei, M. , Szabados, L. and Koncz, C. (2007) Arabidopsis PPP family of serine/threonine phosphatases. Trends Plant Sci. 12, 169–176. 17368080 10.1016/j.tplants.2007.03.003

[pbi70166-bib-0008] Flowers, T.J. , Galal, H.K. and Bromham, L. (2010) Evolution of halophytes: multiple origins of salt tolerance in land plants. Funct. Plant Biol. 37, 604–612 %@ 1445‐4416.

[pbi70166-bib-0009] Geiger, D. , Scherzer, S. , Mumm, P. , Stange, A. , Marten, I. , Bauer, H. , Ache, P. *et al*. (2009) Activity of guard cell anion channel SLAC1 is controlled by drought‐stress signaling kinase‐phosphatase pair. Proc. Natl. Acad. Sci. U. S. A. 106, 21425–21430. 19955405 10.1073/pnas.0912021106 PMC2795561

[pbi70166-bib-0010] Guo, X. , Qin, Q. , Yan, J. , Niu, Y. , Huang, B. , Guan, L. , Li, Y. *et al*. (2015) TYPE‐ONE PROTEIN PHOSPHATASE4 regulates pavement cell interdigitation by modulating PIN‐FORMED1 polarity and trafficking in Arabidopsis. Plant Physiol. 167, 1058–1075. 25560878 10.1104/pp.114.249904 PMC4348754

[pbi70166-bib-0011] Hasegawa, P.M. , Bressan, R.A. , Zhu, J.K. and Bohnert, H.J. (2000) Plant Cellular and Molecular Responses to High Salinity. Annu. Rev. Plant. Physiol. Plant. Mol. Biol. 51, 463–499. 15012199 10.1146/annurev.arplant.51.1.463

[pbi70166-bib-0012] Hou, Y.J. , Zhu, Y. , Wang, P. , Zhao, Y. , Xie, S. , Batelli, G. , Wang, B. *et al*. (2016) Type One Protein Phosphatase 1 and Its Regulatory Protein Inhibitor 2 Negatively Regulate ABA Signaling. PLoS Genet. 12, e1005835. 26943172 10.1371/journal.pgen.1005835 PMC4778861

[pbi70166-bib-0013] Ichimura, K. , Shinozaki, K. , Tena, G. , Sheen, J. , Henry, Y. , (Kazuya Ichimura et al.) MAPK Group , Champion, A. *et al*. (2002) Mitogen‐activated protein kinase cascades in plants: a new nomenclature. Trends Plant Sci. 7, 301–308. 12119167 10.1016/s1360-1385(02)02302-6

[pbi70166-bib-0014] Iliuk, A.B. and Tao, W.A. (2015) Universal non‐antibody detection of protein phosphorylation using pIMAGO. Curr Protoc Chem Biol 7, 17–25. 25727060 10.1002/9780470559277.ch140208 PMC4363923

[pbi70166-bib-0015] Iliuk, A. , Martinez, J.S. , Hall, M.C. and Tao, W.A. (2011) Phosphorylation assay based on multifunctionalized soluble nanopolymer. Anal. Chem. 83, 2767–2774. 21395237 10.1021/ac2000708 PMC3069141

[pbi70166-bib-0016] Jia, W. , Wang, Y. , Zhang, S. and Zhang, J. (2002) Salt‐stress‐induced ABA accumulation is more sensitively triggered in roots than in shoots. J. Exp. Bot. 53, 2201–2206. 12379787 10.1093/jxb/erf079

[pbi70166-bib-0017] Johnson, R.R. , Wagner, R.L. , Verhey, S.D. and Walker‐Simmons, M.K. (2002) The abscisic acid‐responsive kinase PKABA1 interacts with a seed‐specific abscisic acid response element‐binding factor, TaABF, and phosphorylates TaABF peptide sequences. Plant Physiol. 130, 837–846. 12376648 10.1104/pp.001354 PMC166610

[pbi70166-bib-0018] Kamiyama, Y. , Hirotani, M. , Ishikawa, S. , Minegishi, F. , Katagiri, S. , Rogan, C.J. , Takahashi, F. *et al*. (2021) Arabidopsis group C Raf‐like protein kinases negatively regulate abscisic acid signaling and are direct substrates of SnRK2. Proc. Natl. Acad. Sci. U. S. A. 118, e2100073118. 34282011 10.1073/pnas.2100073118 PMC8325330

[pbi70166-bib-0019] Khan, A. , Pan, X. , Najeeb, U. , Tan, D.K.Y. , Fahad, S. , Zahoor, R. and Luo, H. (2018) Coping with drought: stress and adaptive mechanisms, and management through cultural and molecular alternatives in cotton as vital constituents for plant stress resilience and fitness. Biol. Res. 51, 47. 30428929 10.1186/s40659-018-0198-z PMC6234603

[pbi70166-bib-0020] Kong, L. , Cheng, J. , Zhu, Y. , Ding, Y. , Meng, J. , Chen, Z. , Xie, Q. *et al*. (2015) Degradation of the ABA co‐receptor ABI1 by PUB12/13 U‐box E3 ligases. Nat. Commun. 6, 8630. 26482222 10.1038/ncomms9630 PMC4667695

[pbi70166-bib-0021] Krzywinska, E. , Bucholc, M. , Kulik, A. , Ciesielski, A. , Lichocka, M. , Debski, J. , Ludwikow, A. *et al*. (2016) Phosphatase ABI1 and okadaic acid‐sensitive phosphoprotein phosphatases inhibit salt stress‐activated SnRK2.4 kinase. BMC Plant Biol. 16, 136. 27297076 10.1186/s12870-016-0817-1 PMC4907068

[pbi70166-bib-0022] Lee, S.C. , Lan, W. , Buchanan, B.B. and Luan, S. (2009) A protein kinase‐phosphatase pair interacts with an ion channel to regulate ABA signaling in plant guard cells. Proc. Natl. Acad. Sci. U. S. A. 106, 21419–21424. 19955427 10.1073/pnas.0910601106 PMC2795491

[pbi70166-bib-0023] Li, L. , Li, B. , Zhu, S. , Wang, L. , Song, L. , Chen, J. , Ming, Z. *et al*. (2021a) TMK4 receptor kinase negatively modulates ABA signaling by phosphorylating ABI2 and enhancing its activity. J. Integr. Plant Biol. 63, 1161–1178. 33811744 10.1111/jipb.13096

[pbi70166-bib-0024] Li, X. , Yu, B. , Wu, Q. , Min, Q. , Zeng, R. , Xie, Z. and Huang, J. (2021b) OsMADS23 phosphorylated by SAPK9 confers drought and salt tolerance by regulating ABA biosynthesis in rice. PLoS Genet. 17, e1009699. 34343171 10.1371/journal.pgen.1009699 PMC8363014

[pbi70166-bib-0025] Liao, Y.‐D. , Lin, K.‐H. , Chen, C.‐C. and Chiang, C.‐M. (2016) Oryza sativa protein phosphatase 1a (OsPP1a) involved in salt stress tolerance in transgenic rice. Mol. Breed. 36, 1–19.

[pbi70166-bib-0026] Lin, Z. , Li, Y. , Zhang, Z. , Liu, X. , Hsu, C.C. , Du, Y. , Sang, T. *et al*. (2020) A RAF‐SnRK2 kinase cascade mediates early osmotic stress signaling in higher plants. Nat. Commun. 11, 613. 32001690 10.1038/s41467-020-14477-9 PMC6992735

[pbi70166-bib-0027] Liu, Y. , Yan, J. , Qin, Q. , Zhang, J. , Chen, Y. , Zhao, L. , He, K. *et al*. (2020) Type one protein phosphatases (TOPPs) contribute to the plant defense response in Arabidopsis. J. Integr. Plant Biol. 62, 360–377. 31125159 10.1111/jipb.12845

[pbi70166-bib-0028] Ma, Y. , Szostkiewicz, I. , Korte, A. , Moes, D. , Yang, Y. , Christmann, A. and Grill, E. (2009) Regulators of PP2C phosphatase activity function as abscisic acid sensors. Science 324, 1064–1068. 19407143 10.1126/science.1172408

[pbi70166-bib-0029] Mu, C. , Zhou, L. , Shan, L. , Li, F. and Li, Z. (2019) Phosphatase GhDsPTP3a interacts with annexin protein GhANN8b to reversely regulate salt tolerance in cotton (Gossypium spp.). New Phytol. 223, 1856–1872. 30985940 10.1111/nph.15850

[pbi70166-bib-0030] Nolen, B. , Taylor, S. and Ghosh, G. (2004) Regulation of protein kinases; controlling activity through activation segment conformation. Mol. Cell 15, 661–675. 15350212 10.1016/j.molcel.2004.08.024

[pbi70166-bib-0031] Ohta, M. , Guo, Y. , Halfter, U. and Zhu, J.K. (2003) A novel domain in the protein kinase SOS2 mediates interaction with the protein phosphatase 2C ABI2. Proc. Natl. Acad. Sci. U. S. A. 100, 11771–11776. 14504388 10.1073/pnas.2034853100 PMC208833

[pbi70166-bib-0032] Pan, W. , Lin, B. , Yang, X. , Liu, L. , Xia, R. , Li, J. , Wu, Y. *et al*. (2020) The UBC27‐AIRP3 ubiquitination complex modulates ABA signaling by promoting the degradation of ABI1 in Arabidopsis. Proc. Natl. Acad. Sci. U. S. A. 117, 27694–27702. 33077597 10.1073/pnas.2007366117 PMC7959499

[pbi70166-bib-0033] Park, S.Y. , Fung, P. , Nishimura, N. , Jensen, D.R. , Fujii, H. , Zhao, Y. , Lumba, S. *et al*. (2009) Abscisic acid inhibits type 2C protein phosphatases via the PYR/PYL family of START proteins. Science 324, 1068–1071. 19407142 10.1126/science.1173041 PMC2827199

[pbi70166-bib-0034] Qin, Q. , Wang, W. , Guo, X. , Yue, J. , Huang, Y. , Xu, X. , Li, J. *et al*. (2014) Arabidopsis DELLA protein degradation is controlled by a type‐one protein phosphatase, TOPP4. PLoS Genet. 10, e1004464. 25010794 10.1371/journal.pgen.1004464 PMC4091783

[pbi70166-bib-0035] Rodriguez‐Uribe, L. , Higbie, S.M. , Stewart, J.M. , Wilkins, T. , Lindemann, W. , Sengupta‐Gopalan, C. and Zhang, J. (2011) Identification of salt responsive genes using comparative microarray analysis in Upland cotton (Gossypium hirsutum L.). Plant Sci. 180, 461–469. 21421393 10.1016/j.plantsci.2010.10.009

[pbi70166-bib-0036] Rubio, F. , Gassmann, W. and Schroeder, J.I. (1995) Sodium‐driven potassium uptake by the plant potassium transporter HKT1 and mutations conferring salt tolerance. Science 270, 1660–1663. 7502075 10.1126/science.270.5242.1660

[pbi70166-bib-0037] Sharif, I. , Aleem, S. , Farooq, J. , Rizwan, M. , Younas, A. , Sarwar, G. and Chohan, S.M. (2019) Salinity stress in cotton: effects, mechanism of tolerance and its management strategies. Physiol. Mol. Biol. Plants 25, 807–820. 31402811 10.1007/s12298-019-00676-2 PMC6656830

[pbi70166-bib-0038] Soma, F. , Takahashi, F. , Kidokoro, S. , Kameoka, H. , Suzuki, T. , Uga, Y. , Shinozaki, K. *et al*. (2023) Constitutively active B2 Raf‐like kinases are required for drought‐responsive gene expression upstream of ABA‐activated SnRK2 kinases. Proc. Natl. Acad. Sci. U. S. A. 120, e2221863120. 37276398 10.1073/pnas.2221863120 PMC10268249

[pbi70166-bib-0039] Sun, Z. , Feng, Z. , Ding, Y. , Qi, Y. , Jiang, S. , Li, Z. , Wang, Y. *et al*. (2022) RAF22, ABI1 and OST1 form a dynamic interactive network that optimizes plant growth and responses to drought stress in Arabidopsis. Mol. Plant 15, 1192–1210. 35668674 10.1016/j.molp.2022.06.001

[pbi70166-bib-0040] Ubersax, J.A. and Ferrell, J.E., Jr. (2007) Mechanisms of specificity in protein phosphorylation. Nat. Rev. Mol. Cell Biol. 8, 530–541. 17585314 10.1038/nrm2203

[pbi70166-bib-0041] Wang, H. , Chevalier, D. , Larue, C. , Ki Cho, S. and Walker, J.C. (2007) The Protein Phosphatases and Protein Kinases of Arabidopsis thaliana. Arabidopsis Book 5, e0106. 22303230 10.1199/tab.0106 PMC3243368

[pbi70166-bib-0042] Wang, K. , He, J. , Zhao, Y. , Wu, T. , Zhou, X. , Ding, Y. , Kong, L. *et al*. (2018) EAR1 Negatively Regulates ABA Signaling by Enhancing 2C Protein Phosphatase Activity. Plant Cell 30, 815–834. 29618630 10.1105/tpc.17.00875 PMC5969277

[pbi70166-bib-0043] Wang, S. , Guo, J. , Zhang, Y. , Guo, Y. and Ji, W. (2021) Genome‐wide characterization and expression analysis of TOPP‐type protein phosphatases in soybean (Glycine max L.) reveal the role of GmTOPP13 in drought tolerance. Genes Genomics 43, 783–796. 33864615 10.1007/s13258-021-01075-2

[pbi70166-bib-0044] Wang, H. , Wang, Y. , Sang, T. , Lin, Z. , Li, R. , Ren, W. , Shen, X. *et al*. (2023) Cell type‐specific proteomics uncovers a RAF15‐SnRK2.6/OST1 kinase cascade in guard cells. J. Integr. Plant Biol. 65, 2122–2137. 37226855 10.1111/jipb.13536

[pbi70166-bib-0045] Yang, J. , He, H. , He, Y. , Zheng, Q. , Li, Q. , Feng, X. , Wang, P. *et al*. (2021) TMK1‐based auxin signaling regulates abscisic acid responses via phosphorylating ABI1/2 in Arabidopsis. Proc. Natl. Acad. Sci. U. S. A. 118, e2102544118. 34099554 10.1073/pnas.2102544118 PMC8214701

[pbi70166-bib-0046] Yu, F. , Qian, L. , Nibau, C. , Duan, Q. , Kita, D. , Levasseur, K. , Li, X. *et al*. (2012) FERONIA receptor kinase pathway suppresses abscisic acid signaling in Arabidopsis by activating ABI2 phosphatase. Proc. Natl. Acad. Sci. U. S. A. 109, 14693–14698. 22908257 10.1073/pnas.1212547109 PMC3437822

[pbi70166-bib-0047] Yue, J. , Qin, Q. , Meng, S. , Jing, H. , Gou, X. , Li, J. and Hou, S. (2016) TOPP4 Regulates the Stability of PHYTOCHROME INTERACTING FACTOR5 during Photomorphogenesis in Arabidopsis. Plant Physiol. 170, 1381–1397. 26704640 10.1104/pp.15.01729 PMC4775132

[pbi70166-bib-0048] Zhang, L. , Ma, H. , Chen, T. , Pen, J. , Yu, S. and Zhao, X. (2014) Morphological and physiological responses of cotton (Gossypium hirsutum L.) plants to salinity. PLoS One 9, e112807. 25391141 10.1371/journal.pone.0112807 PMC4229235

[pbi70166-bib-0049] Zhang, J. , Qin, Q. , Nan, X. , Guo, Z. , Liu, Y. , Jadoon, S. , Chen, Y. *et al*. (2020) Role of Protein Phosphatase1 Regulatory Subunit3 in Mediating the Abscisic Acid Response. Plant Physiol. 184, 1317–1332. 32948668 10.1104/pp.20.01018 PMC7608174

[pbi70166-bib-0050] Zhao, H. , Li, Z. , Wang, Y. , Wang, J. , Xiao, M. , Liu, H. , Quan, R. *et al*. (2022) Cellulose synthase‐like protein OsCSLD4 plays an important role in the response of rice to salt stress by mediating abscisic acid biosynthesis to regulate osmotic stress tolerance. Plant Biotechnol. J. 20, 468–484. 34664356 10.1111/pbi.13729 PMC8882776

[pbi70166-bib-0051] Zhu, J.K. (2016) Abiotic Stress Signaling and Responses in Plants. Cell 167, 313–324. 27716505 10.1016/j.cell.2016.08.029 PMC5104190

