# Supplementary material for: GhTOPP4aD and GhRAF36 inversely regulate cotton (Gossypium hirsutum) response to ABA and salt stress through reversible phosphorylation of GhABI1
Source: Plant Biotechnol J. 2025 Jun 8;23(9):3561–80. doi: 10.1111/pbi.70166 (PMC12392966; doi:10.1111/pbi.70166)
Supplement: Supplementary file 1 — Figure S1 GhTOPP4aD negatively regulates salt tolerance in cotton. Figure S2 Silencing of GhRAF36 increases the Na+ content under salt stress. Figure S3 GhRAF36 phosphorylates GhABI1 at T124 and S357. Figure S4 GhTOPP4aD interacts with GhABI1. Figure S5 Transcriptome features and relationships among all samples. Figure S6 Analyze the leaves phenotypes of OE/VIGS‐GhTOPP4aD, VIGS‐GhRAF36, and VIGS‐GhABI1 after ABA treatment. Figure S7 Effect of silencing GhABI1 or GhRAF36 on the ABA response of OE‐GhTOPP4aD plants. Figure S8 Effect of silencing GhRAF36 or GhABI1 on the salt response of OE‐GhTOPP4aD plants. Table S1 Partial candidate interaction proteins obtained by screening a yeast two‐hybrid complementary DNA (cDNA) library with GhTOPP4aD. Table S2 Partial candidate interaction proteins obtained by screening a yeast two‐hybrid complementary DNA (cDNA) library with GhRAF36. Table S3 Primers used in this study. Table S4 Gene ontology enrichment analysis of the differentially expressed genes (DEGs) from (Figure 7a). [file PBI-23-3561-s001.docx]

**Supplemental information**

**GhTOPP4aD and GhRAF36 inversely regulate cotton (*Gossypium hirsutum*) response to ABA and salt stress through reversible phosphorylation of GhABI1**

**Fig.S1** GhTOPP4aD negatively regulates salt tolerance in cotton.

**Fig.S2** Silencing of *GhRAF36* increases the Na^+^ content under salt stress.

**Fig.S3** GhRAF36 phosphorylates GhABI1 at T124 and S357.

**Fig.S4** GhTOPP4aD interacts with GhABI1.

**Fig. S5** Transcriptome features and relationships among all samples.

**Fig.S6** Analyze the leaves phenotypes of OE/VIGS-*GhTOPP4aD*, VIGS-*GhRAF36*, and VIGS-*GhABI1* after ABA treatment.

**Fig.S7** Effect of silencing *GhABI1* or *GhRAF36* on the ABA response of OE-*GhTOPP4aD* plants.

**Fig.S8** Effect of silencing *GhRAF36* or *GhABI1* on the salt response of OE-*GhTOPP4aD* plants.

**Supplemental Table 1**. Partial candidate interaction proteins obtained by screening a yeast two-hybrid complementary DNA (cDNA) library with GhTOPP4aD

**Supplemental Table 2**. Partial candidate interaction proteins obtained by screening a yeast two-hybrid complementary DNA (cDNA) library with GhRAF36

**Supplemental Table 3**. Primers used in this study

**Supplemental Table 4**. Gene ontology enrichment analysis of the differentially expressed genes (DEGs) from (Fig. 7a)

**
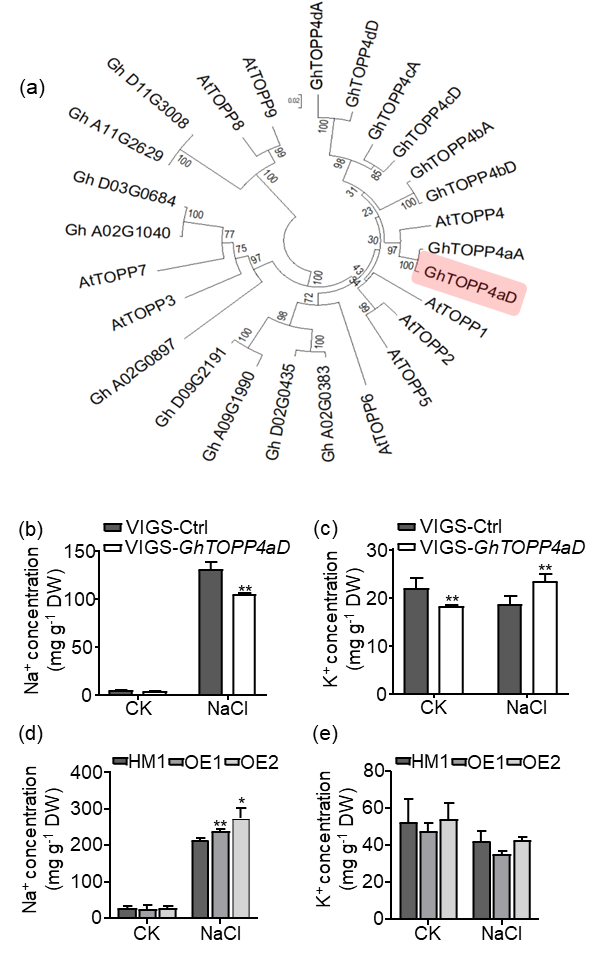
**

Fig.S1 GhTOPP4aD negatively regulates salt tolerance in cotton.

(a) Phylogenetic tree of *TOPP* family in both cotton and Arabidopsis. The amino acid sequences of TOPP in both cotton and Arabidopsis were collected to generate the phylogenetic tree using the neighbor-joining method in MEGA v.6.0. The pink color indicates the gene used in this article, with a corresponding gene ID of Gh_D10G2504. The gene ID of GhTOPP4aA, GhTOPP4bA, GhTOPP4bD, GhTOPP4cA, GhTOPP4cD, GhTOPP4dA and GhTOPP4dD are Gh_A10G2014, Gh_A13G1484, Gh_D13G1801, Gh_A05G0908, Gh_D07G0991, Gh_A03G0379 and Gh_D03G1163, respectively.

(b, c) Na^+^ and K^+^ content in VIGS-Ctrl and VIGS-*GhTOPP4aD*. With 300 mM NaCl treatment for 3 days after VIGS was established, 0.3 g cotton leaves were digested with 1M HCl for 24 h, and the ion accumulation from cotton leaves was measured using an atomic absorption spectrophotometer ‘see the Materials and Methods section’. The data are shown as means ±SD from three independent repeats (n = 3; **, *P* < 0.01, Student’s t-test).

(d, e) Na^+^ and K^+^ content in wild type and OE-*GhTOPP4aD* plants. With 300 mM NaCl treatment for 3 days, 0.3 g cotton leaves were digested with 1M HCl for 24 h, and the ion accumulation from cotton leaves was measured using an atomic absorption spectrophotometer ‘see the Materials and Methods section’. The data are shown as means ±SD from three independent repeats (n = 3; *, *P* < 0.05, **, *P* < 0.01, Student’s t-test).


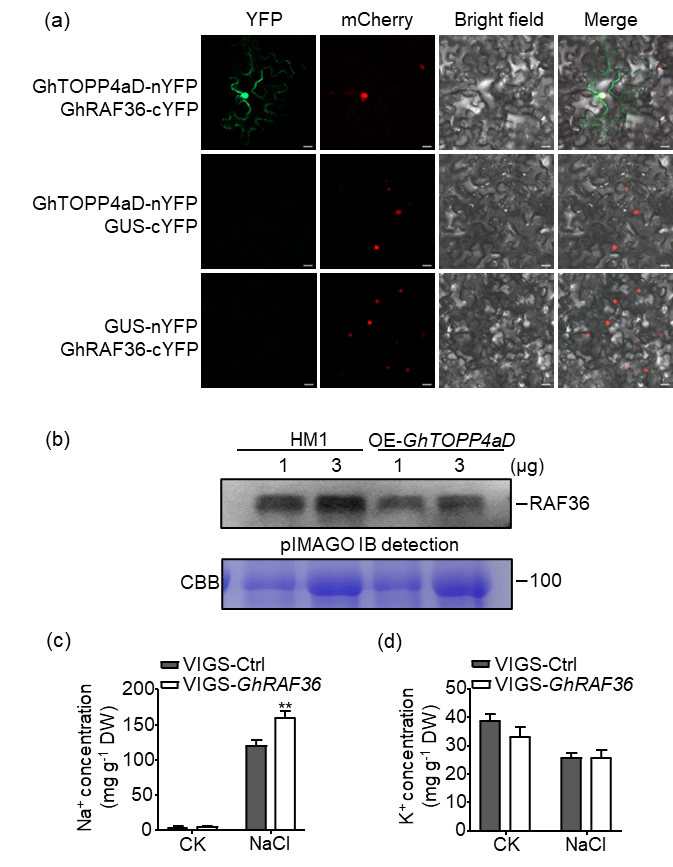


Fig.S2 Silencing of *GhRAF36* increases the Na^+^ content under salt stress.

(a) Detection of the interaction between GhTOPP4aD and GhRAF36 by BiFC assay. β‐glucuronidase (GUS)‐nYFP and GUS‐cYFP were used as negative controls. Scale bars, 50 μm.

(b) GhTOPP4aD inhibits GhRAF36 kinase activity. The total proteins were extracted as kinases from leaves of fourteen-day-old HM1 and OE-*GhTOPP4aD* plants. GhRAF36 as a substrate was incubated with total proteins in kinase reaction buffer for 30 min at 30℃ and then separated by SDS-PAGE.

(c, d) Na^+^ and K^+^ contents in VIGS-Ctrl and VIGS-*GhRAF36* plants. With 300 mM NaCl treatment for 3 days after VIGS was established, 0.3 g cotton leaves were digested with 1M HCl for 24 h, and the ion accumulation from cotton leaves was measured using an atomic absorption spectrophotometer ‘see the Materials and Methods section’. The data are shown as means ±SD from three independent repeats (n = 3; **, *P* < 0.01, Student’s t-test).


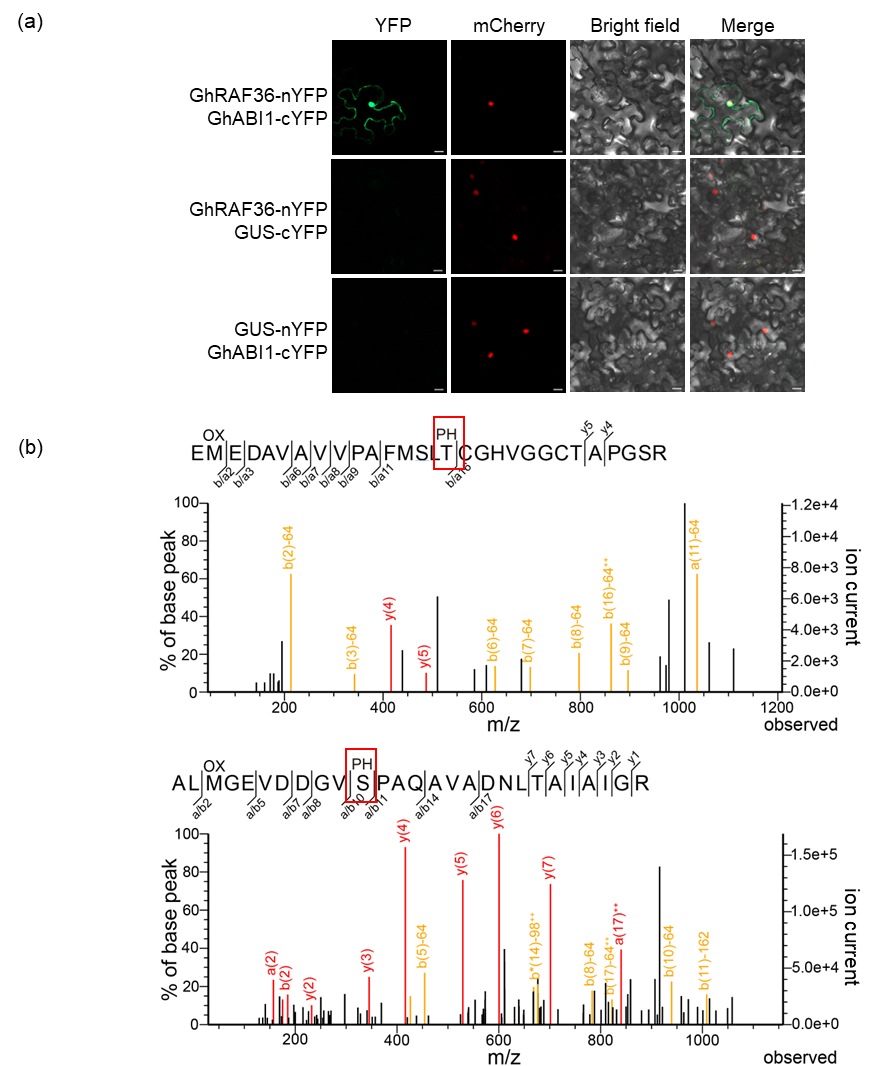


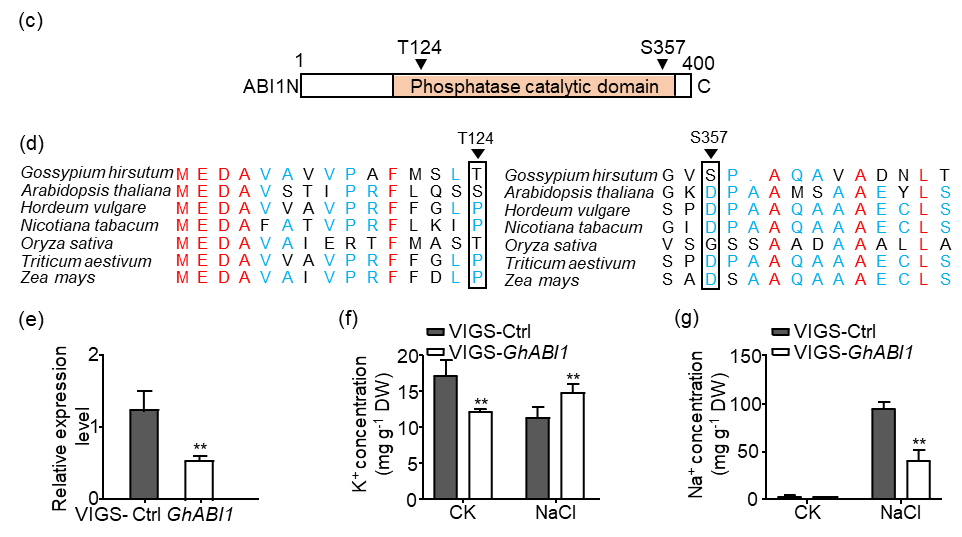


Fig.S3 GhRAF36 phosphorylates GhABI1 at T124 and S357.

(a) Detection of the interaction between GhRAF36 and GhABI1 by BiFC assay. β‐glucuronidase (GUS)‐nYFP and GUS‐cYFP were used as negative controls. Scale bars, 50 μm.

(b) Identification of the phosphorylation sites on GhABI1 by LC-MS/MS assay. GST-GhABI1 was incubated with MBP-GhRAF36 in kinase reaction buffer for 30 min at 30 ℃, followed by trypsin digestion and LC-MS/MS assay.

(c) T124 and S357 are located in the phosphatase catalytic domain of GhABI1.

(d) Sequence alignment of the orthologs of GhABI1 among 7 species. T124 and S357 residue is highlighted in black triangle.

(e) *GhABI1* is speciﬁcally silenced in VIGS-*GhABI1* plants. The leaf samples from VIGS-Ctrl and VIGS-*GhRAF36* cotton seedlings were collected to detect the expression of *GhABI1* without NaCl treatment by RT-qPCR. GhActin9 was used as the internal control. The data are shown as means ±SD from three independent repeats (n = 3; **, *P* < 0.01, Student’s t-test).

(f, g) Na^+^ and K^+^ content in VIGS-Ctrl and VIGS- *GhABI1* plants. With 300 mM NaCl treatment for 3 days after VIGS was established, 0.3 g cotton leaves were digested with 1M HCl for 24 h, and the ion accumulation from cotton leaves was measured using an atomic absorption spectrophotometer ‘see the Materials and Methods section’. The data are shown as means ±SD from three independent repeats (n = 3; **, *P* < 0.01, Student’s t-test).


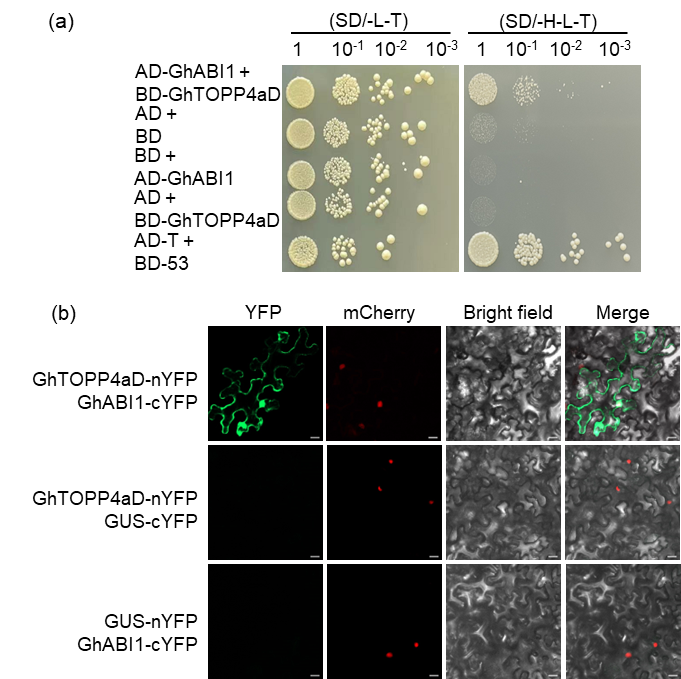


Fig.S4 GhTOPP4aD interacts with GhABI1

(a) Y2H assay showing the interaction between GhTOPP4aD and GhABI1. SD/-L-T, synthetic medium without Trp and Leu; SD/-A-H-L-T, synthetic medium without Trp, Leu, His, Ade. BD and AD were used as empty controls.

(b) Detection of the interaction between GhTOPP4aD and GhABI1 by BiFC assay. β‐glucuronidase (GUS)‐nYFP and GUS‐cYFP were used as negative controls. Scale bars, 50 μm.


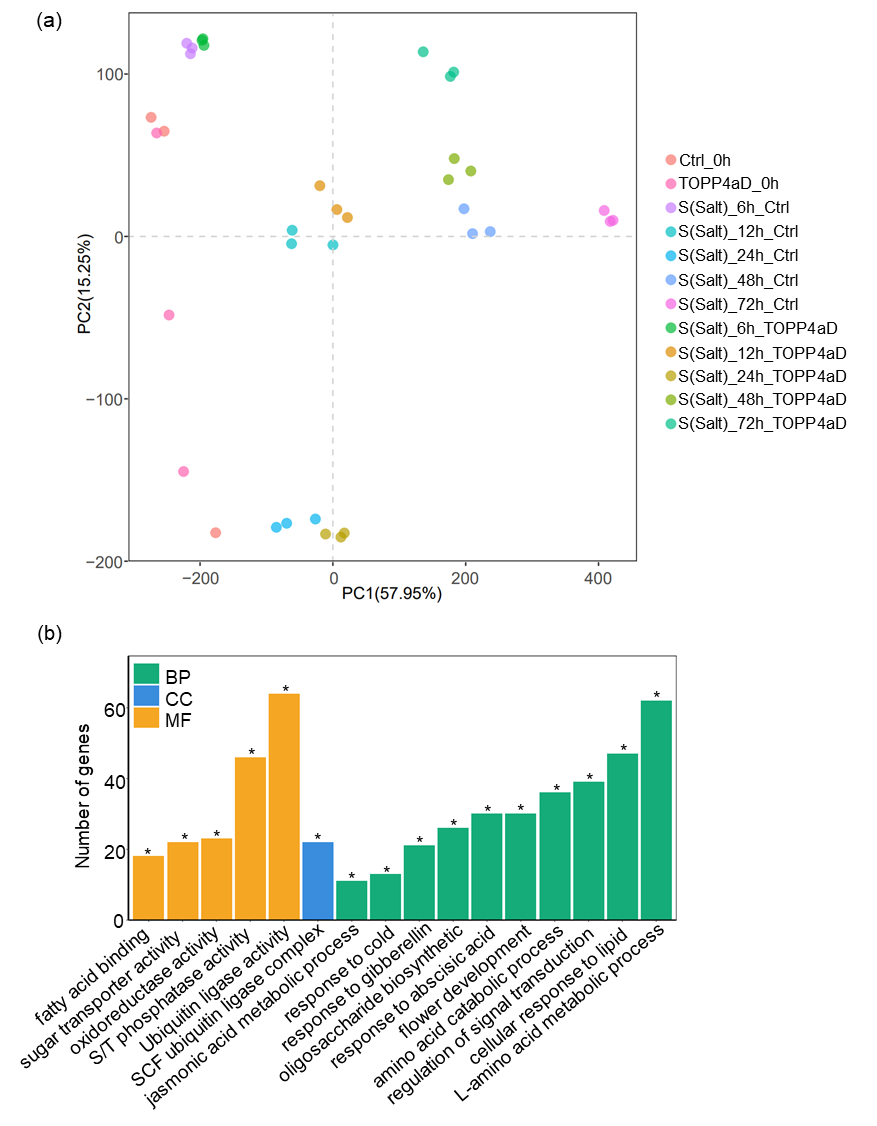


Fig. S5 Transcriptome features and relationships among all samples.

(a) Principal component analysis (PCA) of 36 libraries taken at 6 time points.

(b) Gene ontology enrichment analysis of the differentially expressed genes (DEGs) from (Fig. 5b) (*P*-value < 0.05). BP, Biological Process; CC, Cellular Component; MF, Molecular Function.


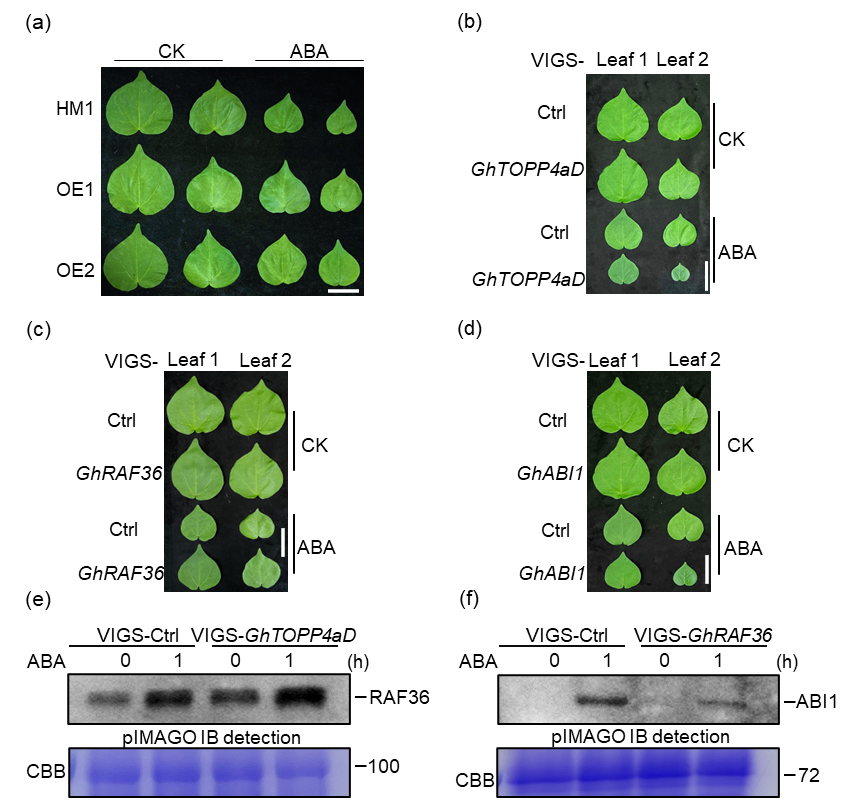


Fig.S6 Analyze the leaves phenotypes of OE/VIGS-*GhTOPP4aD*, VIGS-*GhRAF36*, and VIGS-*GhABI1* after ABA treatment.

(a-d) The leaves Phenotypes of fourteen-day-old OE/VIGS-*GhTOPP4aD*, VIGS-*GhRAF36*, and VIGS-*GhABI1* plants after 7 d of 20 μM ABA treatment. Bar = 3 cm.

(e) Silencing *GhTOPP4aD* enhances ABA-induced kinase activity of GhRAF36. The total proteins were extracted as kinases from leaves of fourteen-day-old VIGS plants with or without ABA treatment. Pre-dephosphorylated GhRAF36 as a substrate was incubated with total proteins in kinase reaction buffer for 30 min at 30℃ and then separated by SDS-PAGE.

(f) Silencing *GhRAF36* inhibits ABA-induced phosphorylation of GhABI1. The total proteins were extracted as kinases from fourteen-day-old VIGS-Ctrl and VIGS-*GhRAF36* cotton leaves with or without ABA treatment. GhABI1 as a substrate was incubated with total proteins in kinase reaction buffer for 30 min at 30℃ and then separated by SDS-PAGE.


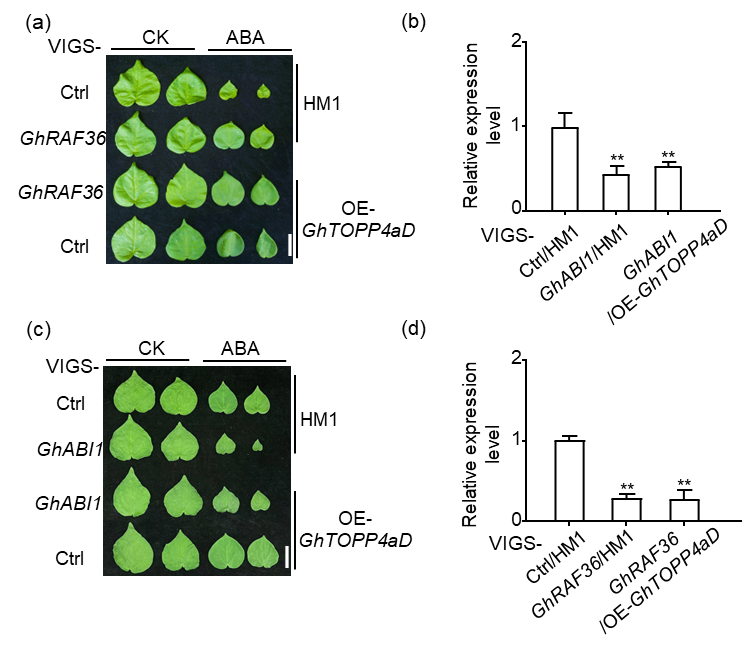


Fig.S7 Effect of *GhABI1* and *GhRAF36* on the ABA response of OE-*GhTOPP4aD* plants.

(a) Silencing *GhRAF36* enhances the ABA insensitivity of OE-*GhTOPP4aD* plants under 20 μM ABA treatment. The first and second leaf were collected for the photograph. Bar = 3 cm.

(b) The silencing efficiency of *GhRAF36* in OE-*GhTOPP4aD* plants. The leaf samples from VIGS plants seedlings were collected to detect the expression of *GhRAF36* by RT-qPCR. GhActin9 was used as the internal control. The data are shown as means ±SD from three independent repeats (n = 3; **, *P* < 0.01, Student’s t-test).

(c) Silencing *GhABI1* compromises the ABA insensitivity of OE-*GhTOPP4aD* plants under 20 μM ABA treatment. The first and second leaves were collected for the photograph. Bar = 3 cm.

(d) The silencing efficiency of *GhABI1* in OE-*GhTOPP4aD* plants. The leaf samples from VIGS plants seedlings were collected to detect the expression of *GhABI1* by RT-qPCR. GhActin9 was used as the internal control. The data are shown as means ±SD from three independent repeats (n = 3; **, *P* < 0.01, Student’s t-test).


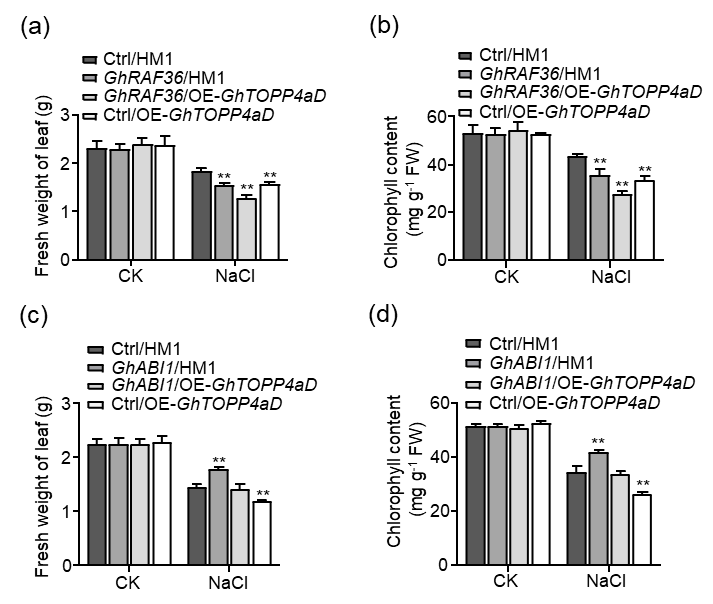


Fig.S8 Effect of silencing *GhRAF36* or *GhABI1* on the salt response of OE-*GhTOPP4aD* plants.

(a, b) The fresh weight of leaves and chlorophyll content without or with NaCl treatment in VIGS-Ctrl/HM1, VIGS-*GhRAF36*/HM1, VIGS-*GhRAF36*/OE-*GhTOPP4aD*, and VIGS-Ctrl/OE-*GhTOPP4aD* without or with NaCl treatment. The data are shown as means ±SD from three independent repeats (n = 3; **, *p* < 0.01, Student’s t-test). The experiments were performed three times with similar results.

(c, d) The fresh weight of leaves and chlorophyll content without or with NaCl treatment in VIGS-Ctrl/HM1, VIGS-*GhABI1*/HM1, VIGS-*GhABI1*/OE-*GhTOPP4aD*, and VIGS-Ctrl/OE-*GhTOPP4aD* without or with NaCl treatment (e). The data are shown as means ±SD from three independent repeats (n = 3; **, *p* < 0.01, Student’s t-test). The experiments were performed three times with similar results.

Supplemental Table 1. Partial candidate interaction proteins obtained by screening a yeast two-hybrid complementary DNA (cDNA) library with GhTOPP4aD

| GENE | GENE ID | FGD description |
| --- | --- | --- |
| *GhLRK* | *Gh_A09G0485* | LEAF RUST 10 DISEASE-RESISTANCE LOCUS RECEPTOR-LIKE PROTEIN KINASE-like 1 |
| *GhRD22* | *Gh_A05G0390* | BURP domain protein RD22 |
| *GhSRF7* | *Gh_A06G1240* | Protein STRUBBELIG-RECEPTOR FAMILY 6 |
| *GhASPG1* | *Gh_A09G1598* | Protein ASPARTIC PROTEASE IN GUARD CELL 1 |
| *GhRAF36* | *Gh_D05G1535* | Group C Raf-like protein kinase RAF36 |
| *GhATM* | *Gh_A05G3117* | Serine/threonine-protein kinase ATM |
| *GhGEM* | *Gh_D10G0977* | GLABRA2 expression modulator |
| *GhABI1* | *Gh_D13G2089* | Protein phosphatase 2C 56,  ABA insensitive 1 |
| *GhUBA2* | *Gh_D03G1351* | Ubiquitin-activating enzyme E12 |
| *GhPOT2* | *Gh_A05G2379* | Potassium transporter 2 |
| *GhGAPC2* | *Gh_D04G0765* | Glyceraldehyde-3-phosphate dehydrogenase 2 |
| *GhATPB* | *Gh_D04G1350* | ATP synthase subunit beta |
| *GhAOS1* | *Gh_A05G2224* | Allene oxide synthase 1 |

Supplemental Table 2. Partial candidate interaction proteins obtained by screening a yeast two-hybrid complementary DNA (cDNA) library with GhRAF36

| *GENE* | *GENE ID* | FGD description |
| --- | --- | --- |
| *GhEF1* | *Gh_D04G0803* | Elongation factor 1-alpha |
| *GhPHOS32* | *Gh_D13G1509* | Universal stress protein PHOS32 |
| *GhNAC72* | *Gh_A04G1303* | NAC domain-containing protein 72 |
| *GhAUX28* | *Gh_D07G2125* | Auxin-induced protein AUX28 |
| *GhSBH2* | *Gh_A03G0871* | Sphinganine C4-monooxygenase 2 |
| *GhABI1* | *Gh_D13G2089* | Protein phosphatase 2C 56,  ABA insensitive 1 |
| *GhERF084* | *Gh_A05G1037* | Ethylene-responsive transcription factor ERF084 |
| *GhCIPK6* | *Gh_A06G0873* | CBL-interacting serine/threonine-protein kinase 6 |
| *GhPCRK1* | *Gh_A10G1831* | Receptor-like protein kinase At5g47070 |
| *GhRAN3* | *Gh_D06G0550* | GTP-binding nuclear protein Ran-3 |
| *GhATPB* | *Gh_D04G1350* | ATP synthase subunit beta |
| *GhLYK3* | *Gh_A02G1137* | LysM domain receptor-like kinase 3 |
| *GhATG18A* | *Gh_A12G0076* | Autophagy-related protein 18a |

Supplemental Table 3. Primers used in this study

| GENE | FGD description |
| --- | --- |
| NptII-F | TCCGGCCGCTTGGGTGGAGAG |
| ptII-R | CTGGCGCGAGCCCCTGATGCT |
| *GhTOPP4aD-*VIGS*-*F | GTGAGTAAGGTTACCGAATTCATGGCGGCTGCGACGGCGC |
| *GhTOPP4aD-*VIGS*-*R | GAGACGCGTGAGCTCGGTACCAGTTCTCTGGGTACTTAAT |
| *GhRAF36*-VIGS-F | GTGAGTAAGGTTACCGAATTCATGTCAGAAGAGGCAAATTCT |
| *GhRAF36*-VIGS-R | GAGACGCGTGAGCTCGGTACCTGAAACCTTCAAGGAACCCA |
| *GhABI1*-VIGS-F | GTGAGTAAGGTTACCGAATTCGTATTCAGTGCGGTTGATTCT |
| *GhABI1*-VIGS-R | GAGACGCGTGAGCTCGGTACCCGTACAACCTCCCACGTG |
| pET28a-*GhTOPP4aD*-F | ATGGGTCGCGGATCCGAATTCATGGCGGCTGCGACGGCGC |
| pET28a-*GhTOPP4aD*-R | GTGGTGGTGGTGGTGCTCGAGCATTTTAGTGGGCATGAACT |
| pET28a-*GhABI1*-F | ATGGGTCGCGGATCCGAATTCATGGAACCCCTACTTGAAGAA |
| pET28a-*GhABI1*-R | GTGGTGGTGGTGGTGCTCGAGTCTTTTTTTCACCGCATTGGA |
| pGEX4T-1-*GhTOPP4aD*-F | GATCTGGTTCCGCGTGGATCCATGGCGGCTGCGACGGCGC |
| pGEX4T-1-*GhTOPP4aD*-R | TCAGTCAGTCACGATGCGGCCCGCCATTTTAGTGGGCATGAACT |
| pGEX4T-1-*GhABI1*-F | GATCTGGTTCCGCGTGGATCCATGGAACCCCTACTTGAAGAA |
| pGEX4T-1-*GhABI1*-R | TCAGTCAGTCACGATGCGGCCCGCTCTTTTTTTCACCGCATTGGA |
| pMALc2X-*GhRAF36*-F | AGGATTTCAGAATTCGGATCCATGTCAGAAGAGGCAAATTCT |
| pMALc2X-*GhRAF36*-R | CAAGCTTGCCTGCAGGTCGACTCATGAGAACTTAGGTTTAGGCA |
| pGADT7-*GhTOPP4aD*-F | TACCAGATTACGCTCATATGATGGCGGCTGCGACGGCGC |
| pGADT7-*GhTOPP4aD*-R | TGCCCACCCGGGTGGAATTCTTACATTTTAGTGGGCATGA |
| pGBKT7-*GhTOPP4aD*-F | CAGAGGAGGACCTGCATATGATGGCGGCTGCGACGGCGC |
| pGBKT7-*GhTOPP4aD*-R | CGACGGATCCCCGGGAATTCTTACATTTTAGTGGGCATGA |
| pGADT7-*GhABI1*-F | TACCAGATTACGCTCATATGATGGAACCCCTACTTGAAGAA |
| pGADT7-*GhABI1*-R | TGCCCACCCGGGTGGAATTCTTATCTTTTTTTCACCGCAT |
| pGBKT7-*GhRAF36*-F | CAGAGGAGGACCTGCATATGATGTCAGAAGAGGCAAATTCT |
| pGBKT7-*GhRAF36*-R | CGACGGATCCCCGGGAATTCTCATGAGAACTTAGGTTTAGGCA |
| pCAMBIA1300*-GhTOPP4aD*-cLUC-F | GTACGCGTCCCGGGGCGGTACCATGGCGGCTGCGACGGCGC |
| pCAMBIA1300*-GhTOPP4aD*-cLUC-R | ACGAAAGCTCTGCAGGTCGACTTACATTTTAGTGGGCATGAAC |
| pCAMBIA1300*-GhABI1*-cLUC-F | GTACGCGTCCCGGGGCGGTACCATGGAACCCCTACTTGAAGAA |
| pCAMBIA1300*-GhABI1*-cLUC-R | ACGAAAGCTCTGCAGGTCGACTTATCTTTTTTTCACCGCAT |
| pCAMBIA1300*-GhRAF36*-nLUC-F | CACGGGGGACGAGCTCGGTACCATGTCAGAAGAGGCAAATTCT |
| pCAMBIA1300*-GhRAF36*-nLUC-R | CGCGTACGAGATCTGGTCGAC |
| pCAMBIA1300*-GhTOPP4aD*-nLUC-F | CACGGGGGACGAGCTCGGTACCATGGCGGCTGCGACGGCGC |
| pCAMBIA1300*-GhTOPP4aD*-nLUC-R | CGCGTACGAGATCTGGTCGACCATTTTAGTGGGCATGAACT |
| pGEX4T-1-*GhABI1*-T124A-F | ATGTCTCTTGCATGTGATCACGTGGGAGGTTGTACGGCTCCTGGTTC |
| pGEX4T-1-*GhABI1*-T124A-R | GTGATCACATGCAAGAGACATGAACGCCGGTACAACGGCGACGGCA |
| pGEX4T-1-*GhABI1*-S357A-F | GATAGAGTTGCACCGGCACAAGCGGTAGCTGATAATCTCACGGCA |
| pGEX4T-1-*GhABI1*-S357A-R | TTGTGCCGGTGCAACTCTATCATCAACTTCGCCCATCAATGCCCTC |
| *GhABI1-*qPCR-F | GGTGACCGCTATTTAAGGC |
| *GhABI1-*qPCR-R | CAACTGCTTCTTCCTATGGCT |
| *GhTOPP4aD -*qPCR-F | CTTATCTCGTCCAACTGC |
| *GhTOPP4aD -*qPCR-R | TCGTAACCATCCTCCAC |
| *GhRAF36-*qPCR-F | TATAAGGAGGAAGCTGTTGC |
| *GhRAF36-*qPCR-R | AGCCCTCTGATAAATACTCTGTT |
| *GhLTI65*-qPCR-F | ACAAAGCCTGAAGAACACCC |
| *GhLTI65*-qPCR-R | AGCTGATCCTTGCGTAGTCG |
| *GhABF3*-qPCR-F | GGAGGCAATCTACAGAGG |
| *GhABF3*-qPCR-R | AATCCAAGAGCTACACCCG |
| *GhABF2-*qPCR-F | TTTGCCCAGGACACTTAG |
| *GhABF2-*qPCR-R | CCCACTGGTATTACTCCCT |
| *GhRD22*-qPCR-F | AGAAACAACAAGGGCTTAC |
| *GhRD22*-qPCR-R | AGGAAATGGCAGACAGG |
